# Titanium Alloy Implants with Lattice Structures for Mandibular Reconstruction

**DOI:** 10.3390/ma17010140

**Published:** 2023-12-27

**Authors:** Khaled M. Hijazi, S. Jeffrey Dixon, Jerrold E. Armstrong, Amin S. Rizkalla

**Affiliations:** 1 School of Biomedical Engineering, Faculty of Engineering, The University of Western Ontario, London, ON N6A 3K7, Canada; 2 Bone and Joint Institute, The University of Western Ontario, London, ON N6G 2V4, Canada; 3 Schulich School of Medicine & Dentistry, The University of Western Ontario, London, ON N6A 5C1, Canada; 4 Division of Oral and Maxillofacial Surgery, Department of Otolaryngology Head and Neck Surgery, Henry Ford Hospital, Detroit, MI 48202, USA; 5 Chemical and Biochemical Engineering, Faculty of Engineering, The University of Western Ontario, London, ON N6A 5B9, Canada

**Keywords:** additive manufacturing, biomaterials, finite element analysis, intraosseous, mandibular biomechanics, mandibular reconstruction, mechanical properties, porous constructs, stress shielding, titanium alloy implants

## Abstract

In recent years, the field of mandibular reconstruction has made great strides in terms of hardware innovations and their clinical applications. There has been considerable interest in using computer-aided design, finite element modelling, and additive manufacturing techniques to build patient-specific surgical implants. Moreover, lattice implants can mimic mandibular bone’s mechanical and structural properties. This article reviews current approaches for mandibular reconstruction, their applications, and their drawbacks. Then, we discuss the potential of mandibular devices with lattice structures, their development and applications, and the challenges for their use in clinical settings.

## 1. The Anatomy and Physiology of the Mandible

**Figure 1:**
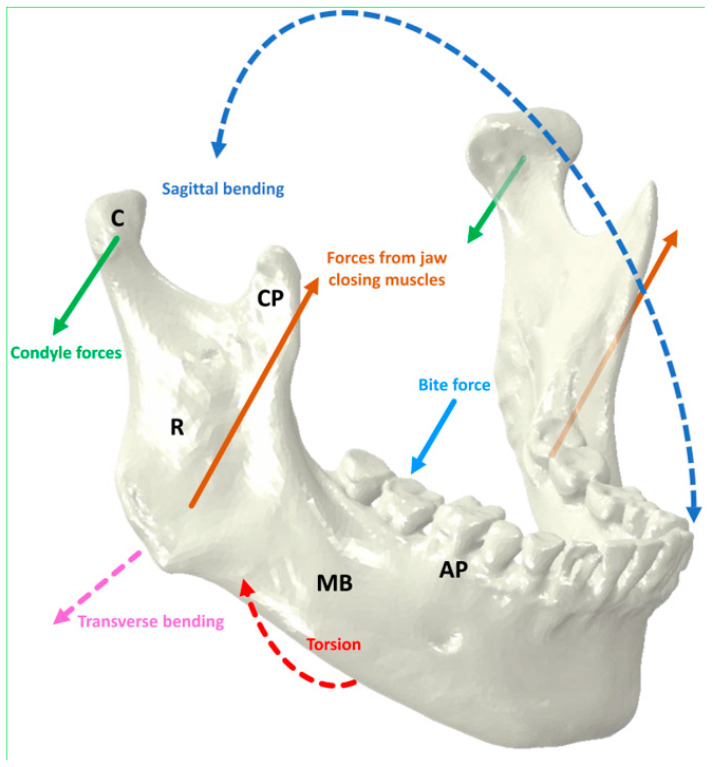
The human mandible includes the mandibular body (MB), two rami (R), two condyles (C), two coronoid processes (CP), and the alveolar process (AP). Solid arrows show loading of the mandible during a unilateral molar bite as described by van Eijden [9]. Distortion of the mandible can be described as a combination of sagittal bending, torsion, and lateral transverse bending, all shown in dashed arrows.

The mandible is the lower jawbone and consists of a body and two rami. The alveolar process anchors the lower teeth, while the condylar processes form part of the temporomandibular joints and the coronoid processes provide muscle attachments (Figure 1). The mandibular bone comprises two layers, a dense layer of cortical bone covering a lighter core of trabecular bone. The thickness of mandibular cortical bone can be between 1 and 4 mm depending on the anatomical location [1,2,3,4,5]. Within the bone, cells specialized in bone remodelling are present. These cells include osteoclasts, osteoblasts, and osteocytes [6]. The mandible’s movement depends on several muscle groups attached to the mandible [7]. The major muscles involved in mastication are the masseter, temporalis, lateral pterygoid, and medial pterygoid. All these muscles, aside from the lateral pterygoid, perform the closing of the jaw. The forces arising from the muscles are balanced by reaction forces acting bilaterally on the temporomandibular joints and teeth [8]. During mastication, forces applied through the teeth and the muscles of mastication cause sagittal bending, transverse bending, and torsion of the mandible [9]. Under these conditions, the mandible typically experiences a combination of tensile loading of the superior and posterior regions and compression loading of the inferior and anterior regions (Figure 1).

The material properties of the mandible are highly dependent on bone density. Variations in the density of bone can be seen in different locations of the mandible and have been shown to affect mechanical strength and stiffness [10,11,12,13]. Young’s modulus values of mandibular cortical bone range between 10 and 31 GPa [11,14,15] and flexural modulus values range between 4.7 and 21 GPa [16]. The trabecular bone has Young’s modulus values that range between 0.003 and 4.5 GPa [11,16,17]. The Young’s modulus of the whole mandible ranges between 0.07 and 3 GPa [16,17], and the flexural modulus ranges between 3.9 and 5.5 GPa [16,18]. The flexural strength of combined mandibular cortical and trabecular bones was determined to reach up to 200 MPa [18]. As in the case for most bones, mandibular bone is highly anisotropic [19].

The static biting forces experienced by mandibles vary according to the location of the applied forces [20,21]. In vivo studies report that the force applied on the teeth and, consequently, on the mandible reaches a maximum of about 300 N in males and 234 N in females under experimental settings [20]. The stress levels from these forces range between 6.5 MPa and 20 MPa during regular muscular activity and chewing [21,22,23,24]. In computer simulations, the stress levels can reach up to 150 MPa during molar clenching, with the highest stress recorded in the mandibular angle and condylar regions [21].

Humans exhibit high variability in the frequency and duration of their chewing activities. Although there have been several attempts to determine the frequency of mastication, only a few studies have examined this parameter in real-life situations. For example, Po et al. [25] conducted a study that monitored the chewing activity of 21 participants using a portable electromyography detector for three days. The participants recorded 2755 chewing activities, translating to approximately 851 bites per day and 311,000 bites yearly. The study revealed that each chewing episode’s mean frequency and duration were 1.5 Hz and 13 s, respectively. Farooq and Sazonov [26] conducted a study in which they tested a system to detect the number of chews during 120 meals with 30 participants. The study found that participants chewed an average of 660 bites per meal at a frequency of 1.53 Hz, resulting in approximately 723,000 chews per year if we assume a human eats three significant meals per day. Other studies, such as that conducted by Chen [27], have estimated that humans can chew up to a million times per year. It is worth noting that although the participants consumed various types of food with varying hardness, the chewing activity was low-force and unlikely to impose excessive stress on the mandible. However, the mandible is consistently stressed due to mastication, swallowing, and, in some cases, parafunctional habits such as clenching and bruxism.

## 2. Mandibular Reconstruction and Fixation Plates

Mandibular reconstruction is a general term encompassing several surgical procedures involved in the repair of the mandible. Mandibular reconstruction is performed when the whole or part of the mandible is destroyed. The goals of mandibular reconstruction are to restore the physical appearance of the lower part of the face, allow for normal eating, optimize speech ability, and allow for normal airflow. Hence, mandibular reconstruction aims to recover the structure of the mandible, provide physical continuity of the mandible, ensure that the reconstructed mandible can withstand masticatory forces, and rehabilitate dental and oral functions [28,29].

Mandibular reconstruction often starts by performing mandibulectomy [30,31], a surgical procedure to remove defective bone parts. Contemporary mandibular reconstruction commonly involves anchoring and bridging the remaining bones using fixation plates while maintaining occlusal function [28,32]. Grafts are applied within the defect to promote bone healing, with the choice of the graft depending on the size of the defect and the condition of the bone. Generally, a non-vascularized graft is used with less severe mandibular defects [33,34,35], whereas more extensive and complex defects require autologously extracted vascularized flaps [29,36,37,38].

Generally, two fracture fixation techniques are applied to mandibular defects during reconstruction [32]. The first is load-bearing fixation, in which the bone is entirely relieved from masticatory loading until healing is complete [39]. This fixation technique is used when fractures occur in severely atrophic bones or where fractures are comminuted (multiple fractures, which form smaller bone pieces). Load-bearing fixation is typically accomplished by using locking reconstruction plates [40,41]. The second fracture fixation technique is load-sharing, which allows the load to be distributed between the hardware and the bone at the fracture site. This technique typically works with more minor or superficial fractures, such as non-complex angle and symphyseal fractures [32,40]. In these cases, the fixation process involves using single miniplates, an arch bar, or lag screw fixation [28,39,40,42]. Fixation plates can be bent to align with the contour of the bone to allow for better facial appearance and for screws to be placed for stabilization [28]. In the case of secondary reconstructions or in cases of tumours that do not allow local fixation plates, complete maxillo-mandibular fixation or an external fixation bridge may be used to maintain proper alignment of the mandible [28,43,44].

## 3. Materials and Techniques Used to Fabricate Fixation Plates

Mandibular fixation devices are made of metals, with the most used materials being stainless steel, pure titanium, and titanium alloys [45,46]. The alloy of titanium that is commonly used is titanium-6 aluminum-4 vanadium (Ti6Al4V) [47,48]. This alloy has a high strength-to-weight ratio [49]. Ti6Al4V is biologically [47] and chemically inert [50,51], largely passive towards corrosion [52,53], and provides an osteoconductive surface which promotes osseointegration [54,55,56,57,58]. Osseointegration is classically defined as the long-term stable fixation of a prosthesis into the adjacent bone tissue [58,59,60].

A combination of formative and subtractive techniques is used conventionally to manufacture surgical implants [51,61]. Formative manufacturing covers techniques that cause a net change of zero in the mass of bulk materials. Some examples include metal casting and forging [62]. Subtractive manufacturing involves the production of a part by removing mass from bulk material, entailing a net loss of material, as in the case of milling, turning, and cutting metals [62]. Formative techniques are first used to establish the implant’s general shape, followed by machining (subtractive manufacturing) of the part to ensure appropriate detailing and finishing [61,62]. Mandibular fixation plates with isotropic and highly controlled material content, geometry, and surface properties can be manufactured using this approach.

## 4. Challenges Associated with the Use of Fixation Plates

Although current approaches for mandibular reconstruction are largely successful [63,64], significant issues need to be addressed. Complications may lead to extended surgical procedures, longer hospital stays and recovery processes, and increased risk of surgical revisions [63,65].

Malunion and malocclusion are widely acknowledged complications in mandibular reconstruction. Malunion can be defined as a bony union formed at an incorrect position [32,66]. Malunion can lead to malocclusions in severe cases, which can cause a loss of occlusal function and facial deformity. Several factors can lead to malunions, including inadequate stabilization, incorrect alignment, inappropriate application or choice of fixation devices, or inadequate bone reduction during surgery [32,66,67]. While malunion defects are detectable early in the healing process, malocclusions are often not recognized until the occlusion is assessed at a late stage [32]. Malunions may require revision surgery.

The non-union of bones is the lack of continuity of bone in a defect post-operation, even after a given healing period has elapsed. High rates of non-union and fibrous union occur in atrophic mandibles with fixation [67]. The non-union of bones can ensue due to poor nutrition, metabolic disturbances, or systemic or localized malignancies and diseases [68,69]. A fibrous union may occur due to fracture instability, infection, or lack of contact between the bone fragments, along with improper application of fixation devices [66]. The non-union of bones appears to occur mainly within the angle region and body of the mandible [69].

Infection is a complication that can arise during mandibular reconstruction, as in any other surgical procedure [70,71]. Regardless of the specific treatment approach used, the risk of infection is present due to bacteria in the oral cavity. These bacteria colonize the site of the defect, or the fixation appliances used during reconstruction, leading to infection-related complications. The risk of infection can be exacerbated by fracture instability and disruption in blood flow [66].

An ongoing challenge of mandibular implants is healing in patients with bone disorders. For example, patients with osteoradionecrosis due to radiation exposure do not respond well to the current clinical techniques of mandibular reconstruction. Osteoradionecrosis can lead to the loosening of hardware post-implantation and other severe side effects [72,73,74].

Autologous bone harvesting has been shown to cause anatomical and physiological disruptions in the donor site. Complications related to autologous grafting include the poor appearance of the skin grafts placed on the donor site, weaknesses in the extension and flexion of extremities, decreased range of motion at the donor site [75,76], difficulties with surgical operations due to logistical limitations, and variations in the amount of bone stock available for the grafting process [63,77,78,79]. Additionally, complications related to blood flow could occur due to the harvesting of arteries from the donor site to assist with the vascularization of grafts [28].

Depending on the fixation method employed, plates are bent to match the desired contour of the bone. A recent clinical study by Zeller et al. [80] showed that the bending of ready-made fixation plates was more likely to cause fractures in these plates when compared to patient-specific fixation plates. The authors attributed this to the bending process inducing stress concentrations within parts of the plates with sharp angles. Moreover, the bending of plates may not provide a similar contour to that of the mandible and is highly dependent on the surgeon’s skill [81]. The bending of fixation plates can also increase the likeliness of fatigue failure.

Another consideration is that current mandibular reconstruction methods do not provide the possibility of matching mechanical properties of the surgical implants to those of adjacent bone. Ti6Al4V is at least three times stiffer than cortical bone [51]. This results in surgical devices and implants bearing a larger portion of the load than adjacent bone, thereby “shielding” the surrounding bone. This mismatch in stiffness results in bones being subjected to inadequate stress levels [35]. Stress shielding leads to impairment of bone remodelling and reduced bone density, which can lead to loosening of the implant [82]. Evidence for stress shielding in mandibular implants is primarily derived from animal studies. Kennady et al. [83,84] reported significantly lower bone volume in primate bones fixed with rigid plating than those without rigid plating. Some clinical studies attributed mandibular bone atrophy to stress shielding directly or indirectly, whereas other clinical studies point towards alternative reasons for rigid implant failure. There appears to be a consensus that the heavier and larger the reconstruction plate, the more likely it is that stress shielding may occur. Zhou et al. [85] detected severe bone resorption in patients with mandibular reconstruction using rigid titanium trays. Many researchers looked at bone height loss after implantation as an indicator of the occurrence of stress shielding. Zoumalan et al. [86] investigated the outcome of fibula free-flap mandibular reconstructions with angular reconstruction plates of 70 patients. These were relatively large reconstruction plates (load-bearing fixation). Zoumalan and coworkers found a marked loss of bone height of approximately 2% about 12 months after implantation. The authors attributed the loss of bone height to stress shielding. Other researchers measured bone height loss after using miniplates. Hidalgo and Pusic [87] conducted a postsurgical investigation of 20 patients who underwent free-flap mandibular reconstruction using miniplates over a period of approximately 10 years. Hidalgo and coworkers found a postoperative bone height loss of approximately 8%. Miniplates (load-sharing fixation) are generally less stiff than large reconstruction fixation plates (load-bearing fixation), and hence it is possible that the greater bone loss associated with load-bearing fixation is due to stress shielding.

According to the mechanostat theory [88,89,90,91], bone modelling is influenced by the strain applied on bone. According to Shen et al. [92], the nature of bone modelling can be matched with four different strain ranges as follows:

1. The disuse state occurs when bone strains fall below 800 µɛ, leading to resorption and atrophy (stress shielding).
2. The adapted state occurs when bone strain is between 800 µɛ and 1500 µɛ, and bone is in a state of homeostasis, with balance between bone formation and resorption.
3. The overload state occurs when the bone deformation is between 1500 µɛ and 15,000 µɛ, and bone modelling and growth occur due to physiologic demand.
4. Pathologic fracture occurs when the strain of bone is beyond 15,000 µɛ, which is associated with the end of elasticity of bone and the initiation of fracture [93].

It can thus be seen that an ideal fixation should allow for some strain to occur, ideally between 800 µɛ and 15,000 µɛ, for homeostasis and growth to occur [90,92,94]. The use of stiff fixation plates might cause the bone to experience less strain, which in turn would promote bone atrophy [95,96].

Design and manufacturing techniques must produce implants with a high level of resolution and sufficient control of geometric and mechanical properties. Conventional manufacturing methods cannot accurately replicate the intricate geometry of human bones [62]. In addition, conventional methods of manufacturing constructs have further disadvantages, including the significant expenditure of time, material, and energy involved in the fabrication process and the susceptibility to oxidation [97]. For these reasons, designers in recent years have adopted additive manufacturing to mimic the geometrical and mechanical properties of bone.

## 5. Additive Manufacturing

With recent developments in computed tomography, magnetic resonance imaging, and medical image processing, researchers can produce digital images and models that accurately represent the geometrical structure of bones [98]. Computer models can be utilized to design implant devices that follow the exact contour and geometry of the original bone structure. Consequently, patient-specific orthopaedic implant models can be produced using additive manufacturing (AM) techniques [42,99,100]. Moreover, computer models can be exported into finite element analysis (FEA) programs to perform mechanical loading simulations and prototyping [21,23,27,92,101]. Such approaches have been used to develop patient-specific conventional fixation plates [42,80,102,103], as well as a multitude of other surgical implants [96,104].

AM, also known as 3D printing, has seen an increase in use by different industries in the past few years, with a global market reaching up to about USD 30 billion in 2022, [100]. AM builds constructs layer by layer. It differs from subtractive or formative manufacturing techniques in that the mass change of the construct during manufacturing is positive [105]. AM provides high precision and accuracy for the fabrication of constructs, making possible the creation of intricate and complex implants that would be difficult to produce using subtractive and equivalent manufacturing techniques [106,107]. Moreover, AM techniques reduce material wastage and manufacturing time [107]. AM is primarily recommended for manufacturing processes where the production volume is low, and design accuracy and intricacy are the main concerns [108,109].

**Figure 2:**
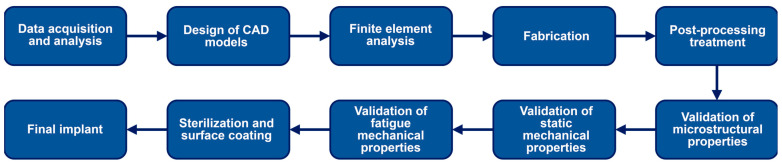
The general process of designing and constructing medical and surgical implants using AM technology.

While various AM technologies have been developed over the years, the general process involved in producing surgical and medical devices using AM technology remains essentially the same (Figure 2). Additive manufacturing requires high levels of precision and computer power to process accurate simulations of FEA models and design intricate prototype models. In addition, the choice of material, input anatomical information accuracy, and output mechanical property requirements are integral to the successful development of implants using AM [108,109].

**Figure 3:**
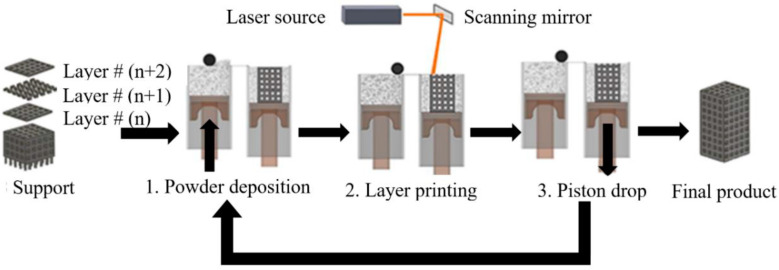
Additive manufacturing process using selective laser melting (SLM) as presented by Jahadakbar et al. [117]. Supports are first built, followed by layer-by-layer fabrication of the constructs. There are two piston-operated chambers, one with the powder and the other where the construct is built. These pistons are shown in brown, while the vertical arrows describe the movement of the parts of the SLM system. 1. The powder is first pushed upward from the powder chamber. The powder is then rolled onto the building chamber using a roller (black circle in the figure). 2. The laser beam is then focused on the powder bed. The layer shape is controlled by managing the laser power and location. 3. Once the layer is completed, the piston in the building chamber is lowered, allowing space for the next layer to be built. This process is repeated until the construct is completed. (This work is licensed under the Creative Commons Attribution 4.0 International License. To view a copy of this license, visit http://creativecommons.org/licenses/by/4.0/ or send a letter to Creative Commons, P.O. Box 1866, Mountain View, CA 94042, USA).

Powder bed fusion techniques, including selective laser melting (SLM), also known as laser powder bed fusion (LPBF), and electron beam melting (EBM), also known as electron powder bed fusion (EPBF), have been of particular interest among researchers [106,110,111]. SLM uses a laser beam to build highly complex constructs from metal powders [112]. During SLM manufacturing, a laser beam melts regions of the powder selectively [113]. After the fabrication of each layer, the build plate lowers by one layer, after which the powder is spread to the next layer, and the laser melting process occurs again [114] (Figure 3). This process repeats until the construct is wholly manufactured [114].

EBM is a technique like SLM, replacing the laser beam with an intensified electron beam. It should be noted that EBM and SLM might be similar in manufacturing, but their output products could be different in mechanical and microstructural properties [115,116].

## 6. Porous Design of Titanium Alloy Constructs

As mentioned above, one disadvantage of using titanium and titanium alloy implants is the considerable difference in stiffness compared to bone. Various methods have been proposed to reduce the stiffness of titanium. The method with the most traction among researchers involves introducing pores into titanium alloy constructs.

Two main approaches have been used to introduce pores. The stochastic design is the earlier developed method in which pores are randomly placed without a specific pattern. Metal foaming [118] and freeze-drying [119] have been used to produce stochastic (random) and close-pore structures. Additive manufacturing techniques have also been used to produce stochastic structures made of Ti6Al4V [120]. The second strategy is the nonstochastic (controlled) porous design, which produces lattice constructs [121]. Nonstochastic porous constructs can only be fabricated using additive manufacturing techniques, as they require a high level of control to produce the periodic patterns of these lattice constructs. Because the porosity and structural properties of lattice constructs are more amenable to control than in stochastic pore structures, the nonstochastic lattice construction process has been the preferred method to produce bone implants. Lattice constructs can reduce the stiffness of constructs to levels comparable to those of cortical and trabecular bones. The geometric properties of the introduced pores, including porosity [122,123], size [124], and shape [125,126], affect the mechanical properties of the lattice constructs.

**Figure 4:**
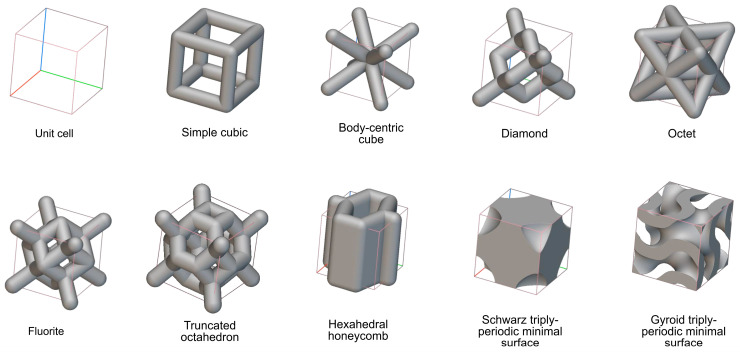
Representative unit cell and cell designs used in studies utilizing nonstochastic lattice constructs. The unit cell size in all these examples was kept consistent at 1 mm, while the feature thickness for all the cells was 250 µm. Complex lattice constructs can be designed by combining different cells.

Nonstochastic constructs consist of unit cells. Unit cell size (UCS) refers to the largest possible diameter of a spherical object that can fit into the unit cell. Features, such as struts or surfaces, are built along the unit cells, with the leftover voids referred to as pores [127]. Feature thickness (FT) refers to the thickness of the features that make up the boundaries of the unit cells, while pore size (PS) refers to the largest possible diameter of a sphere that can fit into the pore when the features are present. Reporting a pore size for some lattice constructs may be inappropriate, as specific unit cells may have complex design features. In such cases, porosity (P), defined as the volume of the void spaces expressed as a percentage of the overall volume of the structure, can be used. Examples of some types of pore design can be seen in Figure 4.

Several recent studies have investigated the mechanical properties of lattice and other porous Ti6Al4V constructs (Table 1). Adding pores to constructs decreases their stiffness and their strength. Therefore, it is crucial to ensure that the lattice properties provide sufficient strength to withstand normal mechanical loading. Traditionally, studies of the mechanical properties of lattice constructs have largely involved compression tests. However, mandibles do not experience pure compressive stress. Therefore, researchers have recently tested other loading regimens. Flexural loading tests such as three-point and four-point bending have been performed on lattice constructs. A summary of some of the results of these tests can be seen in Table 2.

Although many studies have investigated lattice constructs with uniform porosity, recent work has focused on lattice constructs with nonuniform pore geometry. For example, functionally graded porous (FGP) constructs have been designed to match the heterogenous structural or mechanical properties of different locations within bone [3,13]. Porosity gradient FGP designs attempt to match variation in the natural structure of bone [143,144,145]. In topology-optimized FGP designs, the density of the lattice structure is set to match spatial variation in the mechanical properties of bone [143,146]. In both cases, the gradient within FGP designs is ideally continuous to simulate the complex features of bone structure and its mechanical properties [147].

One approach to produce nonuniform lattice designs is to vary pore geometry by changing the unit cell size or to alter strut thickness without changing the unit cell size. Onal et al. [148] found that FGP constructs with a pore size ranging from 400 µm on the outside to 800 µm on the inside could provide higher strength than uniform porous constructs with a strut thickness of 600 µm. Shi et al. [149] and Yang et al. [150] reported on FGP constructs with compressive moduli similar to the elastic moduli of load-bearing bones. Han et al. [151] employed a similar technique to develop FGP titanium alloy constructs with a Schwarz triply periodic minimal surface (TPMS) pore structure. Feature thicknesses varied between 483 and 905 µm, producing compressive modulus values ranging between 0.28 and 0.59 GPa. Song et al. [142] were able to determine the flexural properties of FGP lattice models, where the density of the porous constructs was adapted to the loading on the construct. The results showed that the FGP constructs had superior performance and strength, while minimizing weight, when compared to uniform lattice constructs.

Another approach for the fabrication of nonuniform lattices is to use biphasic designs, with one phase being a lattice structure and the other being a denser lattice or non-lattice shell or core. This approach has received significant acclaim among researchers due to superior static and fatigue strength when compared to fully lattice design concepts. In this regard, Fousova et al. [152] produced constructs with a lattice shell and solid core having a Young’s modulus equivalent to mandibular bone. SLM-built FGP Ti6AL4V constructs, with a triply periodic minimal surface design (gyroid or diamond) cellular structure, were investigated for use as bone scaffolds by Liu et al. [153]. They found that both patterns produced compressive moduli comparable to trabecular bone (≈2.1–3.8 GPa). Using SLM, Xiong et al. [133] built porous constructs with solid cores for potential use in dental implants. The porosities of the constructs ranged between 50 and 70%, and the pore sizes ranged between 420 and 630 µm. The overall compression modulus was between 4 and 11 GPa, within the range of the overall Young’s modulus of mandibular bone. Tüzemen et al. [141] built lattice constructs with functionally graded unit cell sizes and an outer non-lattice skin. These constructs could produce flexural properties comparable to cortical and trabecular bone.

## 7. Bone Growth into Lattice Constructs

Researchers are interested in the potential of lattice SLM-built constructs to allow for bone ingrowth. Bone formation and stabilization around implants have been described in several recent reviews [154,155,156,157]. Xiao et al. [157] and Bai et al. [158] divided the process of bone–implant integration into five phases: blood clotting, immune response, angiogenesis, osteogenesis, and osseointegration. This process is similar to bone healing following fracture except for the lack of callus formation [159].

Immediately following surgical implantation, blood clots form along the implant and bone boundaries [154,157]. Immune cells are recruited, and growth factors are released to stimulate the formation of blood vessels (angiogenesis) and bone (osteogenesis) [157,160,161]. Osteogenesis consists of two processes, osteoinduction and osteoconduction. Osteoinduction is the proliferation and differentiation of mesenchymal stem cells to produce preosteoblasts and then osteoblasts (bone-forming cells) [54]. Osteoconduction is the process by which bone growth is guided along the surface of non-lattice constructs [54,162], or the internal surfaces of lattice constructs [154]. Osseointegration describes the structural and functional integration of the bone and implant along the surfaces in direct contact [59,163]. The extent of osseointegration determines the implant’s long-term functional loading capability [58].

Parameters, such as pore size, shape, porosity, inter-connectivity, and surface roughness influence cell differentiation, bone ingrowth, and ultimately the osseointegration of lattice constructs [126,154,164,165]. In vitro studies have investigated osteogenic cell attachment, proliferation, and differentiation on lattice SLM-built constructs [154,164]. These studies established that pore sizes ranging between 500 and 1000 µm [48,126,166,167] and a porosity of more than 60% [168] are advantageous for osteogenesis. In vivo studies have investigated bone ingrowth into lattice constructs [154,164,169]. A summary of the results from selected in vivo studies can be seen in Table 3. Some studies observed bone ingrowth by measuring the volume of bone as a ratio of the total volume of the specimens (BV/TV) using micro-computed tomography (µCT) [164,170]. Other studies used histological approaches to quantify bone ingrowth [170,171]. Finally, in some studies, mechanical push-out or pull-out analyses were used to determine the level of integration of the bone and the construct, with higher force applied on the specimen indicating higher levels of integration [170,171]. In vivo studies indicated that optimal bone ingrowth occurs at pore sizes ranging between 300 and 900 µm, at porosities ranging between 70 and 84%, and with moderate surface roughness (arithmetic mean height between 1 and 2 µm). In addition, biomimetic coatings, such as hydroxyapatite (HA), can promote the integration of SLM-built titanium constructs.

The time taken for bone ingrowth into lattice titanium alloy constructs is also of interest when designing implants. Integration takes between 6 and 12 weeks to occur in some animal models [168,171,172]. Moreover, 40% bone growth into the lattice constructs was achieved within 6 months in sheep [173]. Implants with porous (sintered) surfaces were shown to take up to 9 months to fully fuse with human bones [174].

**Table 3:** Selected studies investigating bone growth into lattice titanium and Ti6Al4V constructs.

Pore	PS (µm)	P (%)	Ra (µm)	Animal	Key Findings	Reference
Rectangular prism pore	450	-	0.08	Pig	BV/TV = 45% at 60 days Bone density lower than original bone	[170]
Equ tri shell	650	-	-	Beagle	HA-coated shell BA/TA = 50% and porous core BA/TA = 40% at 12 weeks HA-coated specimen had the highest push-out strength by 6 weeks of implantation when compared to non-HA-coated porous specimen HA-coated specimen had the highest BA/TA when compared to non-HA-coated porous specimen	[172]
Equ Tri shell + cir						
Equ Tri shell + cir + HA						
Gyr TPMS	600	-	-	Sheep	The highest push-out force at 12 weeks: SC and Gyr TPMS Highest BV/TV at 12 weeks were found in Gyr, D pyr, and SC Pores with round or quasi-round shape had the largest BV/TV BV/TV at 12 weeks ranged between 60 and 80%	[175]
SC						
Cir						
Tet						
D pyr						
Vor						
SC	700	40	-	Rats	At the same PS and after 4 weeks, P = 70% and P = 90% had significantly higher BV/TV than P = 40% At the same P and after 4 weeks, BV/TV was highest when the nominal PS = 700 µm	[168]
		70				
		90				
	400	70				
	900	70				
Dia Cry	300	62	-	Rabbits	Mature lamellar bone was detected in all the constructs. At 8 weeks, the deepest penetration was in PS = 600 µm At 8 weeks, the detaching (pull-out) force was highest in PS = 600 µm	[171]
	600	66				
	900	64				
SC	700	84	3.33	Rabbits	At 8 weeks, defect bridging was at 80% in all constructs and highest in SLA constructs Mineralized tissue in the original defect was the highest in sandblasted constructs	[176]
SC-Sand			0.94			
SC-SLA			1.16			
Dia	710	68	-	Sheep	CT scans at 3 and 6 months showed significant bone growth. BV/TV at 6 months reached about 40% Mineralized tissue at 6 months made up 38% of the total volume	[173]

Abbreviations: BA/TA—bone area/total area; BV/TV—bone volume/total volume; Cir—circular; Cry—crystal; CT—computed tomography; Dia—diamond; D pyr—double pyramid; Equ tri—equilateral triangle; Gyr TPMS—gyroid TPMS; HA—hydroxyapatite; P—porosity; PS—pore size; Ra—mean arithmetic height of surface roughness; Sand—sandblasted; SC—simple cubic; SLA—sandblasted and chemically etched; Vor—Voronoi. Note: The data reported under key findings were obtained at the maximum time implants were in place. Only in vivo test results are reported.

## 8. Finite Element Analysis in the Development of Lattice Constructs

The gold standard for determining the mechanical properties of SLM-built constructs is to perform uniaxial or multiaxial testing on multiple specimens. However, this step is both costly and time-consuming. Finite element analysis (FEA) has been widely used in the design of mandibular implants and fixation plates as a precursor to real-life pre-clinical mechanical or clinical trials [177]. FEA involves splitting a complex structural model into smaller, less complex parts referred to as finite elements, which can then be resolved through element or nodal analysis [178,179]. As a computer-based numerical technique, FEA enables calculating the mechanical behaviour of structures under various stress conditions [180]. FEA can identify locations of high-stress concentration where implant failure is likely to occur. FEA’s accuracy and precision depend highly on the proper assignment of material properties, boundary and loading conditions, and meshing properties, as well as the calculation method used [177,178,181,182].

When modelling a lattice construct by FEA, three methods can be used. The first involves modelling lattice constructs as solid elements, which is the most used and precise approach for both lattice and non-lattice models [138,183]. This method accurately captures the model’s geometry, including edge effects such as the intersection of struts. It precisely measures the areas where the lattice model is most vulnerable to stress. Potentially any geometry could be modelled using this method. However, using solid elements has several drawbacks, and one of the most disadvantageous is the expensive computational power involved. In addition, the results are highly dependent on the meshing properties. The drawbacks of using solid elements have forced researchers, up until recently, to limit the size of tested models [184]. However, recent developments in the computational power of processors and the introduction of dedicated graphics processing units have opened the possibility of running simulations using more complex lattice properties through solid element modelling [138,185]. Another approach that can be taken is to optimize the size of the mesh elements, where the regions of interest on the models or regions where stress concentrations could be formed would be assigned a finer mesh size and distribution. In contrast, other regions would be given a coarser mesh.

The second method consists of modelling the struts using beams or cables to represent the lattice elements. By omitting the solid thickness of the struts, the meshing process is simplified, leading to faster simulation. Using beam elements only works with beam-based lattice structures and overlooks stress concentrations and edge effects, making it less accurate than solid elements. This technique was used in recent studies, such as that by Song et al. [142]. In that study, beam elements were denoted as “virtual” struts that were used to perform the mechanical simulation analysis. Hence, this technique allows for the modelling of FGP lattice models as well as uniform lattice models. An earlier study by Smith et al. [186] also showed that FEA modelling of constructs using the beam element method produces largely accurate results in models under compression loading.

The third method of modelling lattice constructs in FEA is to use mass and density properties to create representative non-lattice models with the internal density properties associated with lattice models. This technique, known as homogenization, can be used for any lattice and is particularly effective in modelling lattice structures with a high density of pores. However, this method does not assess stress concentration and edge effects, as the exact lattice structure is not provided. Furthermore, this method cannot be used to simulate FGP models. Despite these drawbacks, homogenization may be a good option when the lattice model’s complexity or number of pores makes it impossible to use one of the other two methods. Recent studies, such as the one published by Panettieri et al. [187], assessed the accuracy of homogenization techniques, when compared to modelling lattice constructs as solid elements. Panettieri and co-workers found that homogenized models have the potential to match solid element modeling. Dias et al. [188] adopted homogenization techniques to produce lattice scaffold designs.

FEA has been used previously to study the mechanical properties of the mandible [101,189]. For example, Korioth and Hannam [190] identified the levels of forces and deformation experienced by the mandible during a simulated unilateral molar bite. Examples of prototyping using FEA include studies conducted on scaffolds by Luo et al. [191], who performed mechanical simulations of intercuspal, incisal, and unilateral molar clenching in a mandibular model derived from computed tomography (CT) scans. Other FEA studies have shown that the mandible can experience stress levels ranging between 6.5 and 80 MPa with muscular activity and normal chewing behaviour [21,22,23,24,192].

Ideally, FEA simulations should be validated through mechanical testing of constructs. In this regard, Burton et al. [193] used FEA and mechanical tests to determine the compressive mechanical properties of porous constructs with variations of TPMS pores and simple cubic pores. Another example is the work conducted by Di Caprio et al. [138]. Di Caprio and co-workers used a combination of FEA and mechanical tests to determine the flexural mechanical properties of lattice constructs. They showed that FEA models have the potential to match real-life testing results when strut thicknesses are modified.

Maxillofacial implants and fixation plates have also been studied using FEA modelling. The formation of stress concentrations in bone adjacent to the fixation devices is critical. One example is a study by Ji et al. [194] on the effect of miniplate numbers on stress flow through the adjacent mandibular bone. They found that stress shielding escalates with an increasing number of miniplates, which is expected to affect long-term stability of the miniplates. Another measure of the stability of fixation plates relative to the adjacent bone is the strain level of implants and fixation screws during loading, which can also be estimated through FEA. For example, Zhong et al. [195] conducted a study comparing locking and non-locking, patient-specific mandibular reconstruction plates. The results showed that locking fixation plates exhibited better strength, flexibility, and general safety, as demonstrated by lower von Mises stress, elastic strain, and deformation, than the non-locking fixation plates. Other studies have determined the effect of geometry and orientation of miniplates on stress concentration, with one example being Jesus et al. [196]. In that study, it was found that lambda-shaped plates displayed a more homogeneous stress distribution than the customarily used straight plates. It was also found that fixation devices with patient-specific properties adapted to the bone surface contour are superior to mass-produced fixation plates.

Discrepancies between CAD models and the resulting geometrical properties of lattice constructs have been reported. Horn et al. [137] reported that 3D-printed lattice constructs had strut thicknesses and densities that deviated significantly from the original CAD files. In a recently published work, Di Caprio et al. [138] found that FEA models did not match the flexural properties of their 3D-printed lattice construct counterparts until the strut thickness of the FEA models was reduced by about 20%. These results show that the non-homogeneity of 3D-printed lattice struts and deviations in their geometrical structure should be considered when building FEA models.

Another challenge of FEA analysis is replicating and modelling surface roughness, which is inherent in the 3D printing of Ti6Al4V constructs. Recent attempts to include surface roughness on struts in FEA models are promising. For example, Ghosh et al. [197] assessed the effects of surface texture on the mechanical properties of 3D-printed steel struts. They first analyzed the surface texture geometry and then incorporated it into an FEA model of the struts. A similar study, conducted by Yánez et al. [198], used FEA to analyze the stress concentrations of gyroid TPMS models with different surface roughness levels. Both studies mentioned above showed that stress levels higher than the yield stress of Ti6Al4V can occur due to ridges on the surface. Thus, premature fractures could initiate at the ridges on the surface of the constructs upon application of mechanical loading.

## 9. Lattice-Structured Mandibular Implants with Cage and Crib Designs

Given the benefits of implants with optimized overall geometry and the capability of building lattice structures using additive manufacturing, researchers have been interested in introducing lattice structures into mandibular implants in clinical settings. One design concept that has seen much interest in recent years is the patient-specific intraosseous mandibular implant, with either a cage or a crib structure, to fill the gap formed by mandibular segmentation surgery. This design concept has seen promising clinical results [199,200,201,202,203]. The intraosseous implant design comes with major advantages over the current standard of treatment. While such a design can incorporate bone grafts, it can also function without them, making it a highly versatile option when bone donor site morbidity is a concern. Fatigue fracture risk is reduced as no bending is performed on the implant components during surgery. The technical and surgical complexity is reduced, and the aesthetic outcome is improved due to the matching of implant geometry to the original bone shape [81].

**Figure 5:**
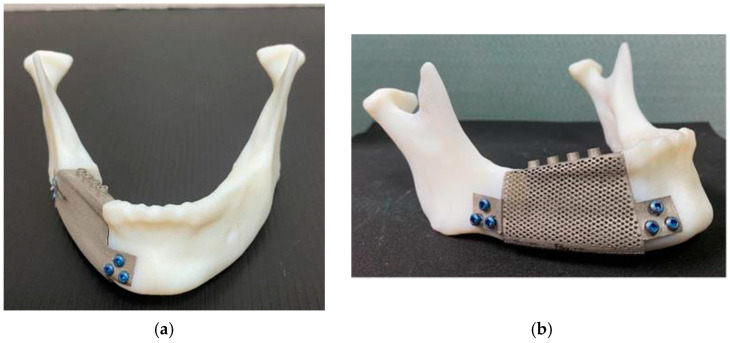
The mandibular implant introduced by Shen et al. [92]. The researchers presented two versions of the implant, the nonporous version of the implant, displayed here from a frontal view (**a**), and the porous version of the implant, displayed here from a side view (**b**). (This work is licensed under the Creative Commons Attribution 4.0 International License. To view a copy of this license, visit http://creativecommons.org/licenses/by/4.0/ or send a letter to Creative Commons, P.O. Box 1866, Mountain View, CA 94042, USA).

In addition to the previously mentioned advantages, the intraosseous implant is able to restore masticatory functions. For example, Shen et al. [92] used FEA and in vitro mechanical tests to assess the design of porous intraosseous (cage) mandibular implants with circular or hexagonal pore shapes and pore sizes of 1 or 2 mm (Figure 5). Such devices were fixed using two small wing plates that attach the implant to the mandible using screws. The mechanical loading and simulations were performed to emulate loading on the mandible through either molar clenching or group function (molars, premolars, and canine). It was reported that porous Ti6Al4V constructs presented higher stresses than nonporous constructs. However, none exhibited von Mises stresses greater than the yield stress of Ti6Al4V. They also found that pores with circular shapes produced lower stress levels than those with hexagonal shapes, indicating that pores with fewer sharp edges may have higher overall resistance to fracture. When molar clenching was simulated, the FEA model detected strain levels in the surrounding bone that would likely lead to bone deformation and, consequently, remodelling [90]. The construct built by the researchers incorporated an abutment base fused with the construct, which could then be used to restore occlusal function. The restoration of masticatory and occlusal function has immense importance as a consideration in the design of mandibular implants.

Peng et al. [23] studied another intraosseous (cage) implant concept. The internal structure of the implant consisted of titanium lattice layers with struts connecting the lattice layers. This design allowed for the use of bone grafts. FEA analysis revealed that von Mises stress concentrations were sufficiently low within the implant. The highest stress was concentrated on the screws attaching the implant to the bone (≈590 MPa). The researchers showed that the proposed design had a stable stress distribution within itself and the adjacent bone, comparable with that seen in healthy mandibular bone, indicating that the model alleviates stress shielding. Through FEA, the researchers tested the design under different degrees of bone ingrowth to assess the stress and strain experienced by the bone and the implant. This design has enormous potential given the stability and general lack of large stress concentrations in the implants; however, this model was not validated by static and dynamic mechanical testing. Nevertheless, results from this study demonstrate the ability of porous lattice constructs to overcome stress shielding.

**Figure 6:**
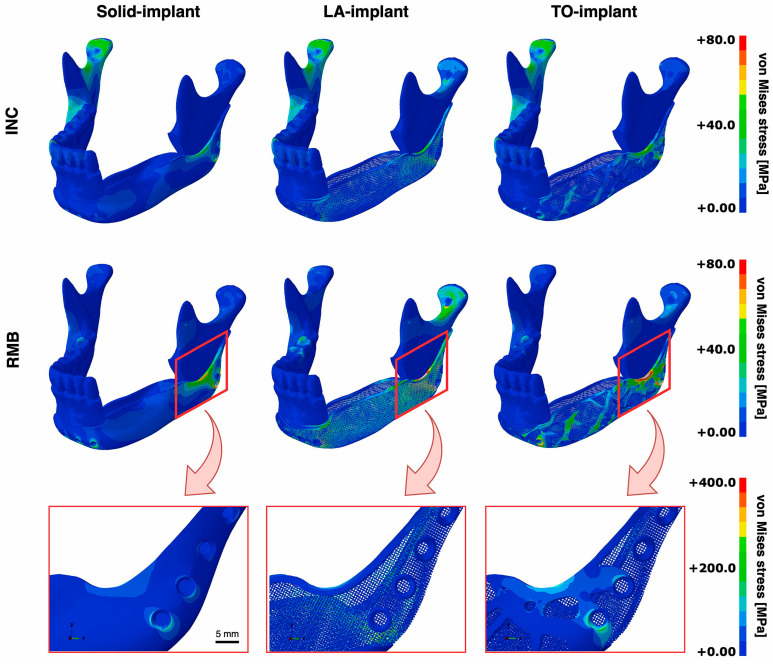
FEA analysis results of the three implants designed by van Kootwijk et al. [81] under incisal clenching (INC) and right molar biting (RMB). The models were fully non-lattice (solid-implant), fully lattice (LA-implant), and topology-optimized (TO-implant) with both lattice and non-lattice parts. The von Mises stresses indicate that no failure is expected within any of the implants, with the highest stress concentrations being located at the angle region of the implant. (Reprinted from reference [81], A. van Kootwijk, V. Moosabeiki, M. Cruz Saldivar, H. Pahlavani, M.A. Leeflang, S. Kazemivand Niar, P. Pellikaan, B.P. Jonker, S.M. Ahmadi, E.B. Wolvius, N. Tümer, M.J. Mirzaali, J. Zhou, A.A. Zadpoor, Semi-automated digital workflow to design and evaluate patient-specific mandibular reconstruction implants, Vol. 132, *Journal of the Mechanical Behavior of Biomedical Materials*, Copyright (2023), with permission from Elsevier.)

A crib design of an intraosseous mandibular implant was proposed by van Kootwijk et al. [81]. The research group utilized the shape of the mandible to design patient-specific mandibular implants using a semi-automated digital workflow. The design utilizes a basket feature, which means that the implant is open in the superior region, making it compatible with bone grafts. Three designs were tested by the authors, one with a nonporous (solid-implant) design, the second with a complete lattice design (LA-implant), and the third design (TO-implant) consisted of a thick set of bars that were optimized topologically to be lightweight, with a finer lattice filling the spaces between the thick bars (Figure 6). Unlike many similar investigations, this study provided both FEA and experimental information to assess the design concept. The researchers also provided a simplified method to experimentally test the mechanical properties of the implant under both static and fatigue loading conditions. The fatigue tests were conducted using constant or incremental cyclic loading within physiological conditions for up to 250,000 cycles at 3 Hz. Results showed that all the implant design models had von Mises stress values that were lower than the yield strength of Ti6Al4V, with stresses concentrated at the angle region of the mandibular implant. All the implants maintained high levels of fatigue strength, with no evidence of failure through the 250,000-cycle loading. The researchers found that the fully lattice design was preferable as it was mechanically compatible with the mandible while maintaining higher porosity and a lower cost of production. This work emphasized the importance of analyzing the mechanical properties of mandibular implants under static and dynamic loading conditions.

Another example of an intraosseous implant was reported by Mommaerts [204], who used porous implants partially covered with shells to restore mandibular defects at the lower part of the mandibular angle and the lateral mandibular border. The design incorporated pore sizes of 500 µm and diamond-shaped pores. Constructs were implanted in 12 patients with aesthetic and malformation complaints. Most patients were satisfied with the procedure. However, it should be noted that the patients were only monitored for up to three months following surgery.

These studies show that using SLM to fabricate porous intraosseous mandibular implants is possible. These patient-specific implants attempted to match the mechanical and geometrical properties of the mandibular bone. Future testing of such design concepts should be conducted using both FEA modelling and mechanical loading analyses.

## 10. Microstructural Imperfections in SLM-Built Ti6Al4V Constructs

There is great potential for using 3D printing in fabricating medical implants. However, the application of additive manufacturing faces several operational and functional challenges. These challenges include microstructural issues and internal defects [205,206,207,208].

The microstructure of Ti6Al4V is known to have two phases, the hexagonal-shaped α phase, which is stabilized by the aluminum, and the body-centric cubic-shaped β phase, which is stabilized by the vanadium [209]. Ideally, an equiphasic α + β microstructure, with α grains and a discontinuous β phase, would provide sufficient ductility and strength. In previous studies, different heat treatments and alloy concentrations were used to vary the microstructure, which gave rise to different mechanical properties [209,210,211]. In SLM-built Ti6Al4V, the microstructure contains prior β grains, within which martensitic α′ platelets are present, giving rise to a relatively high yield strength but low ductility [164,212,213,214]. Yan et al. [211] have shown that lattice Ti6Al4V constructs exposed to hot isostatic pressure (HIP) appear to have both higher compressive strength and higher fracture strain when compared to as-built constructs. HIP involves applying both high temperature and isostatic pressure simultaneously, with the pressure medium being a noble gas like argon. Yan and coworkers also performed microscopic analysis of fracture surfaces in HIP-processed and as-built constructs. They observed a more brittle needle-like α′ martensite in as-built constructs and a more ductile α + β lamellar structure in HIP-processed samples. Ge et al. [210] recently studied SLM-built porous Ti6Al4V constructs. Exposure of these constructs to vacuum annealing converted the microstructure into an equiphasic α + β structure, which improved ductility. Similar work by Yadroitsev et al. [214] showed that the ductility of SLM-built Ti6Al4V constructs increased after annealing, which was explained by the formation of an α + β structure. Thus, the microstructural texture of SLM-built Ti6Al4V constructs has a direct effect on mechanical properties.

Internal defects are gaps formed within the structure of SLM-built constructs. Such internal defects depend largely on the volumetric energy density applied by the laser on the material [215,216]. The equation that governs this process is as follows:

E = P/VHT,(1)

where E is energy density, P is laser power, V is laser scanning velocity (speed), H is hatching spacing, and T is layer thickness. To reduce the number and size of internal defects, the energy density should be optimized, which can be achieved by manipulating the parameters described in Equation (1). The optimization of these parameters for additive manufacturing has been described in multiple studies in the last decade [205,206,216,217,218,219].

Identifying and categorizing internal defects is essential to determine their possible effects on mechanical properties [220]. Defects are typically identified using CT, scanning electron microscopy (SEM), or light microscopy. In general, the three major internal defects that can form on and within SLM constructs are lack-of-fusion, keyhole, and entrapped gas defects [205,208,217]. When the volumetric energy density is too low, lack-of-fusion defects can occur. These defects can be characterized by their larger size (>100 µm) and irregular truncated shape (aspect ratio < 0.5) [205,206]. Lack of fusion occurs when the low energy density causes insufficient melting and fusion of two consecutive layers in the additively manufactured constructs. The irregularity in the geometry of these defects is thought to be caused by an erosion of the substrate along different tracks during the SLM process [207]. This would cause the formation of gaps along the hatch distances of the constructs [208].

When the volumetric energy density is too high, keyhole defects can occur. These defects are similar in size to the lack-of-fusion defects but have a more regular shape (aspect ratio > 0.5) [205,206]. Given the high energy density input, evaporation of the metal can occur, which in turn leads to the keyhole formation within the melt pool [218,221,222]. The cavity forming the keyhole remains open due to vapour pressure, deepening further due to the scattering of heat from the laser [207,223,224]. The keyhole collapses when the laser beam passes, entrapping the vapour into the formed defect.

Gas pores are the smallest internal defects typically seen in SLM constructs. These pores are usually spherical in shape and smaller in size (<100 µm) when compared to the other two kinds of internal defects discussed above [225]. Gas pores are known to occur during the evolution of the melt pool, when it is largely unstable and gas from the environment gets entrapped [226]. It should be noted that gas defects are more likely to occur when the density of the Ti6Al4V powder is low before the 3D-printing process starts [206,227].

Internal defects, particularly lack-of-fusion defects, are known to affect the elastic modulus, strength, elongation, and fatigue resistance of SLM-built nonporous Ti6Al4V constructs [220,228,229,230,231,232,233]. In all the cited studies, the defect volume percentage had to be very high for an appreciable effect on material properties to occur. There is general agreement that internal defects of less than 1% in volume percentage and defect sizes of less than 500 µm will not cause a marked effect on the mechanical integrity of the constructs [212,220]. In addition, some work has shown that defects forming from low volumetric energy density, such as lack-of-fusion defects, can be more detrimental to the static mechanical properties than defects forming due to high volumetric energy density, such as keyhole defects [212,229].

Internal defects take on greater importance when considering lattice-structured porous constructs. This is an active area of investigation among researchers. Salem et al. [227] performed imaging analysis of a series of lattice Ti6Al4V constructs built using SLM to find a range of laser power and scanning speeds that minimized internal defects. Unlike other studies, Salem et al. [227] categorized defects forming due to low energy density input into two categories: lack-of-fusion defects that occur due to high scanning speed together with high laser power and “irregular” defects that occur due to high scanning speed together with low or intermediate laser power. While observing the same internal defects seen in nonporous constructs, Salem et al. [227] indicated that an additional defect, known as sagging, occurs at the junction points of struts in the lattice constructs. These defects occur due to high energy input, which causes excessive molten material to flow downwards under the effect of gravity.

Internal defects in SLM-built Ti6Al4V constructs have been a focus of research over the years. Several methods have been proposed to remove or relieve them. These are further discussed in Section 12 and Section 13 below.

## 11. Fatigue Loading of 3D-Printed Ti6Al4V Constructs

Fatigue properties of 3D-printed Ti6Al4V lattice constructs are critical for their application in clinical settings. The traditional measure of fatigue life in non-lattice Ti6Al4V constructs is the number of cycles that have been applied when the stiffness of the tested construct drops by 90% from its initial stiffness or when the maximum number of cycles (generally between 10^6^ and 10^7^ cycles) is reached [234,235,236,237]. The fatigue life of Ti6Al4V lattice constructs is less than that of non-lattice constructs [238,239]. In lattice structures, fatigue strength and resistance are dependent on the composition and microstructure of the construct [221,240,241,242], surface treatment [243], oxygen content of the surface [244,245], and stresses being applied under fatigue loading [246]. The fatigue behaviour of lattice constructs, normalized to yield strength, has been described to be dependent mostly on the structure’s unit cell shape and surface texture, rather than the density or porosity of the construct [184,247,248].

Several authors have suggested that surface roughness is an important determinant of fatigue properties [198,214,249,250,251]. A rough surface texture has been observed in lattice Ti6Al4V constructs produced using powder bed fusion techniques [210,252,253]. Roughness can occur in SLM constructs due to the adherence of Ti6Al4V particles to the surface, the staircase effect, and the balling effect. Due to thermal diffusion—an outcome of differences in temperature between loose particles and the solidified material—the particles can be partially melted on the surface of the construct [254,255]. In the staircase effect, SLM is unable to produce smooth curvatures due to the layer thickness being too large to capture the curvature, resulting in a rough surface [211,256,257]. The balling effect arises from excessive scanning speed and/or low laser power during 3D printing. These lead to instability in the thermal gradient within the melting pool, which would result in Ti6Al4V particles being melted only partially while also compromising integration with the underlying layer. This causes the particles to form spherical agglomerates along the surface [232,258,259].

The formation of particles on the surface gives rise to alterations in the general geometry of lattice structures. Tüzemen et al. [141], Hernández-Nava et al. [252], and Xiao et al. [253] reported that struts produced using 3D printing techniques had considerable deviations in geometry and thicknesses, driven by surface waviness and roughness. Adherence of powder onto struts has also been described by Song et al. [142], who reported that the thickness of struts in the designed models was less than that in the manufactured constructs. Song and coworkers also found that the FEA models and real-life constructs showed differences in flexural force and displacement values due to inconsistencies in strut thickness. Thus, surface roughness affects the static and fatigue mechanical behaviour of lattice-structured constructs.

It is believed that mechanical failure is caused by large stress concentrations where surface roughness values are highest [198,252,253,260,261]. In their review, Cao et al. [262] and Xiong et al. [239] reported that as-built 3D-printed non-lattice Ti6Al4V constructs are not compatible with fatigue-critical applications without significant post-processing treatments, citing surface roughness as the primary factor. Recent studies have found that the shedding of debris can occur during fatigue loading. Despite constructs remaining visibly intact, debris released during repetitive loading must be considered in biomedical applications as it can induce inflammation and bone resorption [263,264,265].

The role of internal defects, particularly those formed near the surface of lattice constructs, in fatigue fractures has been debated in the literature. Studies on nonporous constructs, such as those conducted by Kasperovich and Hausmann [266], showed that internal defects are the initiators of fatigue cracking. Moreover, it was noted that reducing internal defects using hot isostatic pressure produced better fatigue strength. However, this outcome could be attributed to other factors, such as the microstructure becoming closer to the equiphasic α + β state. Internal defects have been reported to reduce the fatigue life and strength of 3D-printed Ti6Al4V constructs [229,231]. Nevertheless, a considerable body of work suggests that the cause of fatigue failure is stress concentration along surface ridges. According to Dallago et al. [247], under the push–pull fatigue loading of lattice Ti6Al4V constructs, cracks originated from surface ridges rather than internal defects. Similarly, Yánez et al. [198] showed that, while internal defects were detected, they were of sizes smaller than 50 µm and did not appear to be the source of fatigue cracks. Other studies, such as that by Ahmadi et al. [234], suggest that internal defects can cause crack initiation at high stresses and low numbers of loading cycles, whereas surface roughness is the main source of crack initiation at low stresses and high numbers of loading cycles.

A solution proposed to improve the fatigue life of Ti6Al4V lattice constructs is to introduce a nonporous core. Xiong et al. [239] studied Ti6Al4V lattice constructs with dense cores as dental implants both in vitro and in vivo. In vitro, compression fatigue tests were used to compare completely porous constructs to porous constructs with dense cores. Xiong and co-workers found that a porous Ti6Al4V construct with a dense core of about 1.8 mm in diameter and a porosity of 60% exhibited a fatigue strength of about 265 MPa at 10^6^ cycles and an effective fatigue strength of 165 MPa at about 10^5^ cycles. The fatigue strength, found by Xiong and co-workers, for the lattice constructs without a solid core was reduced by about 17 times. van Kootwijk et al. [81] also investigated the effects of using non-lattice parts with porous constructs but found that there were no differences in the mechanical properties. These results were for compressive loading and may not be fully applicable to mandibular implants. Nevertheless, combining nonporous parts in regions of high-stress concentrations within the porous construct could improve the fatigue life of mandibular implants.

## 12. Fabrication Measures to Minimize Structural Imperfections

The optimization of SLM parameters is important to reduce the size and quantity of internal defects, whether constructs are lattice or non-lattice. In addition, the optimization of SLM parameters may avoid melt pool instability, which would also reduce the occurrence of surface roughness. Salem et al. [227] showed that a combination of low laser power and intermediate laser scanning speed produced lattice Ti6Al4V constructs with minimal internal defects. Gong et al. [212] have shown that lower than optimal energy densities and scan speeds are more likely to lead to the formation of lack-of-fusion defects, whereas higher scan speeds and energy densities may promote the formation of keyhole defects. 

Prototyping techniques like FEA can assess different SLM parameters. FEA packages usually include mechanical behaviour analysis, as well as thermal and fluid flow behaviour analysis. The latter analysis is better known as computational fluid dynamics (CFD). Tang et al. [267] used computer simulations to establish that fusion defects can be avoided by optimizing layer thickness, hatch spacing, and laser scanning speed. Khairallah et al. [268] also used simulations to show that increasing laser power can reduce the formation of keyhole pores in 3D-printed metal constructs. Xiang et al. [259] used computer simulations to assess the formation of lack-of-fusion defects and surface roughness on 3D-printed constructs with different printing parameters, such as beam speed, layer thickness, and hatch spacing. Kasperovich and Hausmann [266] recommended a moderate scanning speed and a larger laser spot size to reduce internal defects. In addition, melting and subsequent cooling rates significantly affected internal defects [206,219]. Entrapped gas defects could be reduced by using a sufficient amount and density of Ti6Al4V powder during the printing process [269,270].

Regardless of the fabrication measures taken to minimize internal defects and surface roughness, optimization of SLM parameters cannot completely remove structural imperfections.

## 13. Post-Processing Treatments to Improve Mechanical Properties and Minimize Structural Imperfections

Post-processing treatments have been employed to reduce structural imperfections and to improve the mechanical properties of Ti6Al4V constructs. 

Heat treatments are routinely conducted on constructs post-manufacturing to relieve residual stresses formed during the fabrication process [271,272,273]. One such treatment, better known as annealing, is used to homogenize the microstructure of SLM-built constructs [272,273,274]. This approach was reported by Yuan et al. [274], who tested lattice constructs with simple cubic pore topology and strut thicknesses of around 500 µm. They applied static and dynamic compression loading. They showed that annealing changed the microstructure of the constructs through the formation of coarse α lamellae with a large thickness:length ratio. This change increased the ductility of the constructs and, in turn, ramped up fatigue endurance to about 60% of the yield stress, which is well within the endurance limit for dense lattice and non-lattice Ti6Al4V structures.

However, traditional heat treatments do not reduce internal defects or surface roughness. One treatment that addressees the issue of internal defects is hot isostatic pressure (HIP). HIP utilizes both heat (≈900 °C) and isostatic pressure (≈100 MPa), which causes shrinking and densification of the construct from all directions [275,276,277,278]. HIP has been shown to relieve Ti6Al4V constructs from internal defects, increasing their strength [279,280]. In addition, Kasperovich and Hausmann [266] showed that HIP produces an effect on the microstructure of SLM-built constructs similar to that produced by annealing, with the internal structure becoming an equiphasic α + β state. Hence, it can be seen that HIP can replace annealing while also reducing internal defects. A solution to deal with sub-surface pores is to apply laser shock peening, which improves the fatigue properties of Ti6Al4V [281,282]. It also decreases the roughness of SLM-manufactured Ti6Al4V constructs by about 50% and increases their fatigue life by ten-fold [283].

There is a wide array of post-processing surface treatments. In general, mandibular implants must exhibit high fatigue strength while maintaining a level of surface roughness that promotes bone cell attachment and proliferation [284]. A common surface treatment applied to SLM-built constructs is sandblasting or grit-blasting [284]. In both cases, abrasive particles of sand or grit are applied under high pressure to the surface of the construct, removing irregularities and unmelted particles and thus reducing roughness. However, a disadvantage of this technique is the potential for introducing bacteria into the construct that can hinder bone growth and lead to infection [285]. Mechanical polishing is the simplest procedure to reduce surface roughness. However, mechanical polishing does not achieve the uniform smoothing of complex structures [286], making it unsuitable for use in lattice constructs. Other surface treatments for lattice constructs have been considered. These include electropolishing, where the SLM-built construct, as the anode, is immersed in an electrolyte with a cathode and placed under direct current [287]. Under direct current, a reduction reaction occurs on the SLM-built construct, which uniformly removes surface irregularities [52,287,288,289,290]. Another method to reduce surface roughness is chemical etching, which involves the use of acidic etchants to smoothen partially welded powder particles. A major advantage of this technique is that it allows for the removal of surface roughness from within the lattice constructs, unlike most other methods which target only the outer surfaces [291]. On the other hand, chemical etching has been shown to be largely nonuniform in its treatment of surface roughness in SLM-built constructs [292]. 

Given the advantages and disadvantages of post-processing treatments, some researchers advocate for the use of multiple treatments for SLM constructs. For example, Dong et al. [287] and Pyka et al. [293] showed that electropolishing can reduce the surface roughness of Ti6Al4V lattice constructs when combined with etching. Another approach that Ahmadi et al. [260] suggested was a combination of HIP treatment, sandblasting, and a moderate amount of chemical etching to reduce the surface roughness. This combination increased the compression and fatigue strength of lattice constructs as well as their ductility. Similarly, Jamshidi et al. [284] have shown that a combination of HIP, sandblasting, wet centrifugal polishing, and chemical etching produces non-lattice constructs with enhanced ductility and improved tensile and fatigue strength when compared to as-built constructs. In addition, Jamshidi and coworkers showed that chemical etching, when combined with other treatments, enhances cellular adhesion and proliferation on the surface of SLM-built constructs. It should be noted that there is evidence that HIP can affect other surface roughness treatments, such as grit-blasting [294]. Hence, any additional surface modifications should be performed after HIP is applied.

Although all these solutions have shown promise in reducing surface roughness and internal defects in lattice constructs, they also come with the disadvantage of decreasing strut thickness or altering the structure of struts. For example, Ahmadi et al. [260] found that chemical etching can significantly reduce strut thickness. This could be challenging when dealing with lattice structures having small strut thicknesses.

## 14. Challenges with the Clinical Application of 3D-Printed Mandibular Implants

Several challenges are hindering the introduction of permanent, patient-specific, lattice-structured mandibular implants. One challenge is reconstruction of the mandibular condyle. The condyle is the focus of movement of the mandible, and damage in that region can affect the normal range of motion. Such damage can cause difficulties in mastication and deep breathing, and deficiency in mandibular height [295]. Current treatment choices include using implants made of alloplastic materials (such as copper or titanium alloys) [296,297]. Some researchers suggest combining such implants with remodelled autogenous grafts [298]. However, these options are either prone to complications or not universally suitable for patients. In addition, titanium devices for condylar reconstruction are associated with the risk of erosion [296], which would cause implant displacement and pain [299]. In addition, lattice titanium alloys are expected to be highly susceptible to fracture due to friction. Hence, lattice-structured titanium alloy implants are not suitable for condylar reconstruction.

Challenges in the use of lattice titanium alloy implants extend to post-implantation functionality. One issue is the time of healing. There are few clinical studies on the time it takes for bone to grow into porous Ti6Al4V constructs. A few studies reported that porous mandibular implants appeared to be stabilized in patients within three months [300,301,302]. In vivo, animal models suggest that this process could take up to 6 months [173]. During bone healing, the issue of septic loosening may arise. Septic loosening is due to infection introduced at the point of implantation or by underlying infections [303,304]. One solution may be to introduce surface coatings onto mandibular implants, containing anti-bacterial agents and/or growth factors to minimize inflammation and stimulate bone ingrowth [303].

While several concept designs of intraosseous mandibular implants have been proposed over the years, these devices have not undergone extensive clinical experimentation. Nevertheless, recent small-scale studies have shown promising outcomes. Xia et al. [104] studied 10 oncology patients treated with customized Ti6Al4V implants without bone grafts. Results were compared to 10 patients who received vascularized bone grafts and conventional fixation. Compared to patients who underwent conventional treatment, patients from the titanium implant group showed improvements on several fronts, including mandibular contour symmetry, oral and masticatory function, range of mouth opening, and pain related to the temporomandibular joint. The paper did not mention pore topology and properties, but the authors indicated that porosity levels were such that the structural and mechanical properties of the implants matched those of the mandible. 

While showing great potential, the use of additive manufacturing in producing clinically viable mandibular implants can be hindered by several challenges. The clinical challenges might require further innovative solutions to these problems, which should be the focus of future research.

## 15. Gaps of Knowledge

While a great amount of work has been published on designing and manufacturing lattice constructs using 3D printing techniques, this topic is still a subject of active research. Developments in design and testing are needed to ensure that a standardized and methodical workflow is established for the efficient fabrication of safe and effective constructs.

It should be noted that there is a considerable gap in the literature regarding the flexural fatigue properties of Ti6Al4V lattice constructs. This is thought to be due to the complexity of three- and four-point loading regimens used to study fatigue properties [246]. Most of the studies reported in the literature dealt with compression fatigue testing [239,248,260,305]. However, during mastication, mandibles are subjected to flexural loading, tensile loading at the inferior border, and compressive loading in the alveolar process [9,306]. Mandibular reconstruction plates and implants must endure similar loading conditions. Assessment of the flexural fatigue properties of 3D-printed lattice-structured Ti6Al4V implants would be beneficial to understand their failure mechanism. Flexural loading is more complex than the classically studied compression or push–pull fatigue loading. Flexural loading may be a more appropriate way of investigating mandibular mechanics and the response of lattice Ti6Al4V constructs to static and fatigue loading. Thus, focusing on flexural fatigue properties of lattice constructs should be considered a high priority for future work.

Furthermore, FEA models of lattice Ti6Al4V constructs need to be refined, taking into consideration the presence of internal defects and surface roughness. Although attempts have been made to create such FEA models recently, studies were limited by the computational power available. Improved FEA models would allow for faster and more accurate pre-manufacturing prediction of the mechanical properties of constructs. In addition, if FEA modelling is to become standard for the production of patient-specific implants, then improved FEA models should be available for use without the need for large expenditures of time and computational power.

Further investigation of approaches for alleviating microstructural defects from 3D-printed lattice constructs is required. At this point, there is no standard approach for post-processing heat treatment and surface treatment of lattice constructs. A technique that could be utilized involves computer simulation of 3D-printing parameters using CFD techniques, which have shown potential in determining thermal gradient differences during 3D printing.

The development of lattice structures for oral and maxillofacial applications shows promise for improving aesthetics and mechanical performance. However, an optimal design for mandibular implants that does not require bone grafts would be ideal. Such devices could provide patients with a solution requiring fewer surgical hours and no donor site morbidity. Optimization would require investigation into promoting bone ingrowth and reducing the possibility of postsurgical infection.

## 16. Conclusions

There continues to be considerable interest in the use of 3D-printed Ti6Al4V lattice-structured constructs for mandibular reconstruction. Additive manufacturing techniques can now be used for the fabrication of Ti-alloy implants. Developing and designing patient-specific mandibular implants would open the door for more efficient mandibular reconstruction. New intraosseous porous implants for bone defects would reduce surgical times and revision surgeries, provide clinicians with additional treatment options, and accelerate healing and restoration of function. Future studies are needed to establish optimal pore geometry for lattice constructs with improved mechanical properties and extended service lives.

## Figures and Tables

**Table 1:** Selected studies on the static loading of SLM-built lattice constructs.

Lattice	LT	P (%)	PS (µm)	FT (µm)	UCS (mm)	E (GPa)	σu (MPa)	σy (MPa)	Reference
SC	T	64	500	300	0.8	42.0	-	-	[128]
		70	600	300	0.9	28.6			
		84	500	150	0.7	22.6			
		88	600	150	0.8	16.1			
		93	600	100	0.7	12.4			
SC	C	93	-	670	4.0	3.0	16	16	[129]
		93		670	4.0	1.8	9	9	
		94		670	4.0	1.8	9	9	
		93		500	3.0	3.0	15	14	
Dia	C	20	-	600	-	11.8	200	-	[130]
		26		360		9.6	150		
		44		360		5.0	146		
		51		840		9.3	96		
		56		1000		8.3	228		
		65		840		4.2	69		
		66		600		7.7	185		
		73		840		1.6	82		
		75		600		1.4	52		
		75		600		1.3	47		
		76		600		1.2	49		
		78		600		0.4	26		
		82		840		0.4	19		
		89		200		0.2	10		
		90		600		0.2	8		
		91		360		0.3	9		
		93		360		0.1	4		
Dia TPMS	C	-	500	-	1.0	0.6	-	-	[131]
Gyr TPMS			200		1.0	0.6			
Neov TPMS			350		1.0	0.6			
Stochastic	C	87	830	210	-	1.7	550	-	[132]
Dia	C	66	631	283	-	4.7	171	127	[133]
Hex		67	643	285		3.8	163	110	
Dia-S		51	636	283		10.1	420	350	
Hex-S		53	643	285		11.0	537	424	
Schw TPMS	C	25	138	768	-	58.0	-	520	[134]
		42	282	635		44.0		325	
		64	569	552		22.3		160	
Diagonal	C	50	-	1669	5.0	10	200	-	[135]
Rhombic				1317		20	200	-	
Dia	C	80		200	-	1.2	36	-	[136]
		76		250		2.0	57		
		73	650	300		3.0	86		
		68		350		3.8	109		
		66		400		5.2	140		

Abbreviations: BCC—body-centric cubic; C—compression; Dia—diamond; E—modulus; EBM—electron beam melting; FT—feature thickness; Gyr—gyroid; Hex—hexagonal; LT—loading type; σy—yield strength; σu—ultimate strength; Neov—neovious; P—porosity; PS—pore size; S—support; SLM—selective laser melting; Sch—Schwarz; SC—simple cubic; T—tensile; TPMS—triply periodic minimal surface; UCS—unit cell size. Note that unavailable modulus and strength values were obtained from published force, displacement, and stiffness values or estimated from the provided graphs.

**Table 2:** Selected studies on the flexural static loading of additively manufactured lattice constructs.

Lattice	AMT	LT	P (%)	FT (µm)	UCS (mm)	E (GPa)	σF (MPa)	Reference
RDod	EBM	4PB	80	454	3	5.7	60	[137]
			70	575	3	7.7	63	
			60	722	3	10.7	84	
			80	905	6	1.7	27	
			70	1153	6	5.6	75	
			60	1408	6	12.5	132	
			80	1361	9	2.4	32	
			70	1732	9	6.6	73	
			60	2096	9	14.1	120	
OT	EBM	3PB	-	1000	-	2.5	60	[138]
OT (OS)						27.6	376	
OT (OS; SL = 200 mm)	EBM	3PB	-	600	6	18.4	312	[139,140]
OT (SL = 120 mm)						12.5	312	
OT (OS; SL = 45 mm)						2.04	237	
FGP–Dia (OS)	SLM	3PB	79	300	1.8–2.2	1.41	100	[141]
FGP–Dia (OS)			61	500		2.81	500	
FGP–Dia (OS)			41	700		3.82	780	
FGP–SC (OS)			85	300		0.38	25	
FGP–SC (OS)			75	500		1.15	80	
FGP–SC (OS)			63	700		2.42	270	
FGP–Oct (OS)			84	300		0.66	40	
FGP–Oct (OS)			74	500		1.68	140	
FGP–Oct (OS)			63	700		2.66	320	
FGP–BCC	SLM	3PB		260–810	2	6.4	300	[142]
BCC				200		0.8	66	

Abbreviations: 3PB—three-point bending; 4PB—four-point bending; AMT—additive manufacturing technique; BCC—body-centric cubic; C—compression; Dia—diamond; E—modulus; EBM—electron beam melting; FGP—functionally graded porosity; FT—feature thickness; LT—loading type; OT—octet truss; OS—outer skin; P—porosity; PS—pore size; RDod—rhombic dodecahedron; SC—simple cubic; SLM—selective laser melting; σ_F_—flexural strength; TPMS—triply periodic minimal surface; UCS—unit cell size. Note that unavailable modulus and strength values were obtained from published force, displacement, and stiffness values or estimated from the provided graphs.

## Data Availability

Not applicable.

## References

[1] Heibel H., Alt K.W., Wachter R., Bahr W. (2001). Kortikalisdicke am Unterkiefer unter besonderer Berücksichtigung der Miniplattenosteosynthese Morphometrische Analyse an Sektionsmaterial. Oral Maxillofac. Surg..

[2] Katranji A., Misch K., Wang H.L. (2007). Cortical bone thickness in dentate and edentulous human cadavers. J. Periodontol..

[3] Schwartz-Dabney C.L., Dechow P.C. (2002). Edentulation alters material properties of cortical bone in the human mandible. J. Dent. Res..

[4] Santos I.G., Ramos de Faria F., da Silva Campos M.J., de Barros B.A.C., Rabelo G.D., Devito K.L. (2023). Fractal dimension, lacunarity, and cortical thickness in the mandible: Analyzing differences between healthy men and women with cone-beam computed tomography. Imaging Sci. Dent..

[5] Khairy S.G., Mahaini L. (2015). Determination of Buccal Cortical Bone Thickness for Mini-Screws Placement in Horizontal Growth Type Patients by Cone Beam Computed Tomography. J. Dent. Health Oral Disord. Ther..

[6] Maciel G.B.M., Maciel R.M., Danesi C.C. (2023). Bone cells and their role in physiological remodeling. Mol. Biol. Rep..

[7] Wexler A.M., Taub P.J., Patel P.K., Buchman S.R., Cohen M.N. (2015). Anatomy of the Head and Neck. Ferraro’s Fundamentals of Maxillofacial Surgery.

[8] Faulkner M.G., Hatcher D.C., Hay A. (1987). A three-dimensional investigation of temporomandibular joint loading. J. Biomech..

[9] van Eijden T.M.G. (2000). Biomechanics of the mandible. Crit. Rev. Oral Biol. Med..

[10] Andani M.T., Shayesteh Moghaddam N., Haberland C., Dean D., Miller M.J., Elahinia M. (2014). Metals for bone implants. Part 1. Powder metallurgy and implant rendering. Acta Biomater..

[11] Nagasao T., Miyamoto J., Kawana H. (2009). Biomechanical evaluation of implant placement in the reconstructed mandible. Int. J. Oral Maxillofac. Implants.

[12] Shapurian T., Damoulis P.D., Reiser G.M., Griffin T.J., Rand W.M. (2006). Quantitative Evaluation of Bone Density Using the Hounsfield Index. Int. J. Oral Maxillofac. Implants.

[13] Schwartz-Dabney C.L., Dechow P.C. (2003). Variations in cortical material properties throughout the human dentate mandible. Am. J. Phys. Anthropol..

[14] Bujtár P., Sándor G.K.B., Bojtos A., Szűcs A., Barabás J. (2010). Finite element analysis of the human mandible at 3 different stages of life. Oral Surg. Oral Med. Oral Pathol. Oral Radiol. Endodontology.

[15] Seong W.J., Kim U.K., Swift J.Q., Heo Y.C., Hodges J.S., Ko C.C. (2009). Elastic properties and apparent density of human edentulous maxilla and mandible. Int. J. Oral Maxillofac. Surg..

[16] Odin G., Savoldelli C., Bouchard P.O., Tillier Y. (2010). Determination of Young’s modulus of mandibular bone using inverse analysis. Med. Eng. Phys..

[17] Lakatos E., Magyar L., Bojtar I. (2014). Material Properties of the Mandibular Trabecular Bone. J. Med. Eng..

[18] Vitins V., Dobelis M., Middleton J., Limbert G., Knets I. (2003). Flexural and creep properties of human jaw compact bone for FEA studies. Comput. Methods Biomech. Biomed. Eng..

[19] Augat P., Link T., Lang T.F., Lin J.C., Majumdar S., Genant H.K. (1998). Anisotropy of the elastic modulus of trabecular bone specimens from different anatomical locations. Med. Eng. Phys..

[20] Ferrario V., Sforza C., Serrao G., Dellavia C., Tartaglia G. (2004). Single tooth bite forces in healthy young adults. J. Oral Rehabil..

[21] Pinheiro M., Alves J.L. (2015). The feasibility of a custom-made endoprosthesis in mandibular reconstruction: Implant design and finite element analysis. J. Craniomaxillofac. Surg..

[22] Gholampour S., Gholampour H., Khanmohammadi H. (2019). Finite element analysis of occlusal splint therapy in patients with bruxism. BMC Oral Health.

[23] Peng W.M., Cheng K.J., Liu Y.F., Nizza M., Baur D.A., Jiang X.F., Dong X.T. (2021). Biomechanical and Mechanostat analysis of a titanium layered porous implant for mandibular reconstruction: The effect of the topology optimization design. Mater. Sci. Eng. C Mater. Biol. Appl..

[24] Wong R.C., Tideman H., Merkx M.A., Jansen J., Goh S.M. (2012). The modular endoprosthesis for mandibular body replacement. Part 1: Mechanical testing of the reconstruction. J. Craniomaxillofac. Surg..

[25] Po J.M., Kieser J.A., Gallo L.M., Tesenyi A.J., Herbison P., Farella M. (2011). Time-frequency analysis of chewing activity in the natural environment. J. Dent. Res..

[26] Farooq M., Sazonov E. (2016). Automatic Measurement of Chew Count and Chewing Rate during Food Intake. Electronics.

[27] Chen L. (2012). Finite Element Analysis of the Stress on the Implant-Bone Interface of Dental Implants with Different Structures. Finite Element Analysis—New Trends and Developments.

[28] Bak M., Jacobson A.S., Buchbinder D., Urken M.L. (2010). Contemporary reconstruction of the mandible. Oral Oncol..

[29] Batstone M.D. (2018). Reconstruction of major defects of the jaws. Aust. Dent. J..

[30] Hanasono M.M., Militsakh O.N., Richmon J.D., Rosenthal E.L., Wax M.K. (2013). Mandibulectomy and Free Flap Reconstruction for Bisphosphonate-Related Osteonecrosis of the Jaws. JAMA Otolaryngol.–Head Neck Surg..

[31] Schrom T., Bast F., Knipping S. (2019). Partial mandibulectomy without bony reconstruction in patients with oropharyngeal or mouth cancer. Contemp. Oncol./Współczesna Onkol..

[32] Pickrell B.B., Serebrakian A.T., Maricevich R.S. (2017). Mandible Fractures. Semin. Plast. Surg..

[33] Marechek A., AlShare A., Pack S., Demko C., Quereshy F.A., Baur D. (2019). Nonvascularized Bone Grafts for Reconstruction of Segmental Mandibular Defects: Is Length of Graft a Factor of Success?. J. Oral Maxillofac. Surg..

[34] Ren Z.H., Fan T.F., Zhang S., Wu H.J. (2020). Nonvascularized Iliac Bone Reconstruction for the Mandible without Maxillofacial Skin Scarring. J. Oral Maxillofac. Surg..

[35] Pogrel M.A. (2020). The Concept of Stress Shielding in Non-vascularized Bone Grafts of the Mandible-A Review of Two Cases. J. Oral Maxillofac. Surg..

[36] Hidalgo D.A. (1989). Fibula free flap: A new method of mandible reconstruction. Plast. Reconstr. Surg..

[37] Farwell D.G., Kezirian E.J., Heydt J.L., Yueh B., Futran N.D. (2006). Efficacy of small reconstruction plates in vascularized bone graft mandibular reconstruction. Head Neck.

[38] Kearns M., Ermogenous P., Myers S., Ghanem A.M. (2018). Osteocutaneous flaps for head and neck reconstruction: A focused evaluation of donor site morbidity and patient reported outcome measures in different reconstruction options. Arch. Plast. Surg..

[39] Ellis E., Miles B.A. (2007). Fractures of the mandible: A technical perspective. Plast. Reconstr. Surg..

[40] Yadav A., Bonanthaya K., Panneerselvam E., Manuel S., Kumar V.V., Rai A. (2021). Principles of Internal Fixation in Maxillofacial Surgery. Oral and Maxillofacial Surgery for the Clinician.

[41] Harjani B., Singh R.K., Pal U.S., Singh G. (2012). Locking v/s non-locking reconstruction plates in mandibular reconstruction. Natl. J. Maxillofac. Surg..

[42] Kreutzer K., Steffen C., Koerdt S., Doll C., Ebker T., Nahles S., Flugge T., Heiland M., Beck-Broichsitter B., Rendenbach C. (2022). Patient-Specific 3D-Printed Miniplates for Free Flap Fixation at the Mandible: A Feasibility Study. Front. Surg..

[43] Militsakh O.N., Wallace D.I., Kriet J.D., Girod D.A., Olvera M.S., Tsue T.T. (2004). Use of the 2.0-mm Locking Reconstruction Plate in Primary Oromandibular Reconstruction after Composite Resection. Otolaryngol. Head Neck Surg..

[44] Ung F., Rocco J.W., Deschler D.G. (2002). Temporary intraoperative external fixation in mandibular reconstruction. Laryngoscope.

[45] Barros S.E., Vanz V., Chiqueto K., Janson G., Ferreira E. (2021). Mechanical strength of stainless steel and titanium alloy mini-implants with different diameters: An experimental laboratory study. Prog. Orthod..

[46] Riviș M., Roi C., Roi A., Nica D., Văleanu A., Rusu L.-C. (2020). The Implications of Titanium Alloys Applied in Maxillofacial Osteosynthesis. Appl. Sci..

[47] Sidambe A.T. (2014). Biocompatibility of Advanced Manufactured Titanium Implants—A Review. Materials.

[48] Warnke P.H., Douglas T., Wollny P., Sherry E., Steiner M., Galonska S., Becker S.T., Springer I.N., Wiltfang J., Sivananthan S. (2009). Rapid prototyping: Porous titanium alloy scaffolds produced by selective laser melting for bone tissue engineering. Tissue Eng. Part C Methods.

[49] Meier B., Warchomicka F., Petrusa J., Kaindl R., Waldhauser W., Sommitsch C. (2023). High Temperature Tensile Strength of TI6AL4V Processed by L-PBF—Influence of Microstructure and Heat Treatment. BHM Berg-und Hüttenmännische Monatshefte.

[50] Tengvall P., Lundström I. (1992). Physico-chemical considerations of titanium as a biomaterial. Clin. Mater..

[51] Jackson M.J., Kopac J., Balazic M., Bombac D., Brojan M., Kosel F. (2016). Titanium and Titanium Alloy Applications in Medicine. Surgical Tools and Medical Devices.

[52] Rahman Z.U., Pompa L., Haider W. (2014). Influence of Electropolishing and Magnetoelectropolishing on Corrosion and Biocompatibility of Titanium Implants. J. Mater. Eng. Perform..

[53] Schiff N., Grosgogeat B., Lissac M., Dalard F. (2002). Influence of fluoride content and pH on the corrosion resistance of titanium and its alloys. Biomaterials.

[54] Albrektsson T., Johansson C. (2001). Osteoinduction, osteoconduction and osseointegration. Eur. Spine J..

[55] Albrektsson T., Meredith N., Wennerberg A., Branemark P.I., Tolman D.E. (1999). Osseointegration in Craniofacial Reconstruction.

[56] Branemark P.I., Tolman D.E. (1998). Osseointegration in Craniofacial Reconstruction.

[57] Higuchi K.W. (2000). Osseointegration or osteointegration?. Oral Surg Oral Medicine Oral Pathol. Oral Radiol. Endod.

[58] Jayesh R.S., Dhinakarsamy V. (2015). Osseointegration. J. Pharm. Bioallied Sci..

[59] Branemark P.I. (1983). Osseointegration and its experimental background. J. Prosthet. Dent..

[60] Granstrom G. (2007). Craniofacial osseointegration. Oral Dis..

[61] Abellán-Nebot J.V., Siller H.R., Vila C., Rodríguez C.A. (2012). An experimental study of process variables in turning operations of Ti–6Al–4V and Cr–Co spherical prostheses. Int. J. Adv. Manuf. Technol..

[62] DeBoer B., Nguyen N., Diba F., Hosseini A. (2021). Additive, subtractive, and formative manufacturing of metal components: A life cycle assessment comparison. Int. J. Adv. Manuf. Technol..

[63] Kakarala K., Shnayder Y., Tsue T.T., Girod D.A. (2018). Mandibular reconstruction. Oral Oncol..

[64] Keller E.E., Tolman D., Eckert S. (1998). Endosseous implant and autogenous bone graft reconstruction of mandibular discontinuity: A 12-year longitudinal study of 31 patients. Int. J. Oral Maxillofac. Implants.

[65] Vignesh U., Mehrotra D., Howlader D., Singh P.K., Gupta S. (2019). Patient Specific Three-Dimensional Implant for Reconstruction of Complex Mandibular Defect. J. Craniofacial Surg..

[66] Perez D., Ellis E. (2020). Complications of Mandibular Fracture Repair and Secondary Reconstruction. Semin. Plast. Surg..

[67] Ellis E. (1996). Complications of rigid internal fixation for mandibular fractures. J. Cranio-Maxillofac. Trauma.

[68] Mathog R.H., Toma V., Clayman L., Wolf S. (2000). Nonunion of the mandible: An analysis of contributing factors. J. Oral Maxillofac. Surg..

[69] Bochlogyros P.N. (1985). Non-union of fractures of the mandible. J. Maxillofac. Surg..

[70] Radwan D., Mobarak F. (2018). Plate-related complications after mandibular reconstruction: Observational study osteotomy. Egypt. J. Oral Maxillofac. Surg..

[71] Seol G.-J., Jeon E.-G., Lee J.-S., Choi S.-Y., Kim J.-W., Kwon T.-G., Paeng J.-Y. (2014). Reconstruction plates used in the surgery for mandibular discontinuity defect. J. Korean Assoc. Oral Maxillofac. Surg..

[72] Buchbinder D., Currivan R.B., Kaplan A.J., Urken M.L. (1993). Mobilization regimens for the prevention of jaw hypomobility in the radiated patient: A comparison of three techniques. J. Oral Maxillofac. Surg..

[73] Ichimura K., Tanaka T. (1993). Trismus in patients with malignant tumours in the head and neck. J. Laryngol. Otol..

[74] Marx R.E. (1983). Osteoradionecrosis: A new concept of its pathophysiology. J. Oral Maxillofac. Surg..

[75] Daniels T.R., Thomas R., Bell T.H., Neligan P.C. (2005). Functional Outcome of the Foot and Ankle After Free Fibular Graft. Foot Ankle Int..

[76] Yim K.K., Wei F.C. (1994). Fibula osteoseptocutaneous flap for mandible reconstruction. Microsurgery.

[77] Ling X.F., Peng X. (2012). What Is the Price to Pay for a Free Fibula Flap? A Systematic Review of Donor-Site Morbidity following Free Fibula Flap Surgery. Plast. Reconstr. Surg..

[78] Momoh A.O., Yu P., Skoracki R.J., Liu S., Feng L., Hanasono M.M. (2011). A Prospective Cohort Study of Fibula Free Flap Donor-Site Morbidity in 157 Consecutive Patients. Plast. Reconstr. Surg..

[79] Pare A., Bossard A., Laure B., Weiss P., Gauthier O., Corre P. (2019). Reconstruction of segmental mandibular defects: Current procedures and perspectives. Laryngoscope Investig. Otolaryngol..

[80] Zeller A.N., Neuhaus M.T., Weissbach L.V.M., Rana M., Dhawan A., Eckstein F.M., Gellrich N.C., Zimmerer R.M. (2020). Patient-Specific Mandibular Reconstruction Plates Increase Accuracy and Long-Term Stability in Immediate Alloplastic Reconstruction of Segmental Mandibular Defects. J. Maxillofac. Oral Surg..

[81] van Kootwijk A., Moosabeiki V., Saldivar M.C., Pahlavani H., Leeflang M.A., Kazemivand Niar S., Pellikaan P., Jonker B.P., Ahmadi S.M., Wolvius E.B. (2022). Semi-automated digital workflow to design and evaluate patient-specific mandibular reconstruction implants. J. Mech. Behav. Biomed. Mater..

[82] Sumitomo N., Noritake K., Hattori T., Morikawa K., Niwa S., Sato K., Niinomi M. (2008). Experiment study on fracture fixation with low rigidity titanium alloy. J. Mater. Sci. Mater. Med..

[83] Kennady M.C., Tucker M.R., Lester G.E., Buckley M.J. (1989). Stress shielding effect of rigid internal fixation plates on mandibular bone grafts. A photon absorption densitometry and quantitative computerized tomographic evaluation. Int. J. Oral Maxillofac. Surg..

[84] Kennady M.C., Tucker M.R., Lester G.E., Buckley M.J. (1989). Histomorphometric evaluation of stress shielding in mandibular continuity defects treated with rigid fixation plates and bone grafts. Int. J. Oral Maxillofac. Surg..

[85] Zhou L.B., Shang H.T., He L.S., Bo B., Liu G.C., Liu Y.P., Zhao J.L. (2010). Accurate reconstruction of discontinuous mandible using a reverse engineering/computer-aided design/rapid prototyping technique: A preliminary clinical study. J. Oral Maxillofac. Surg..

[86] Zoumalan R.A., Hirsch D.L., Levine J.P., Saadeh P.B. (2009). Plating in microvascular reconstruction of the mandible: Can fixation be too rigid?. J. Craniofac. Surg..

[87] Hidalgo D.A., Pusic A.L. (2002). Free-flap mandibular reconstruction: A 10-year follow-up study. Plast. Reconstr. Surg..

[88] Pahr D.H., Reisinger A.G. (2020). A Review on Recent Advances in the Constitutive Modeling of Bone Tissue. Curr. Osteoporos. Rep..

[89] Meslier Q.A., Shefelbine S.J. (2023). Using Finite Element Modeling in Bone Mechanoadaptation. Curr. Osteoporos. Rep..

[90] Frost H.M. (1987). Bone “mass” and the “mechanostat”: A proposal. Anat. Rec..

[91] Piccinini M., Cugnoni J., Botsis J., Ammann P., Wiskott A. (2016). Numerical prediction of peri-implant bone adaptation: Comparison of mechanical stimuli and sensitivity to modeling parameters. Med. Eng. Phys..

[92] Shen Y.W., Tsai Y.S., Hsu J.T., Shie M.Y., Huang H.L., Fuh L.J. (2022). Biomechanical Analyses of Porous Designs of 3D-Printed Titanium Implant for Mandibular Segmental Osteotomy Defects. Materials.

[93] Biewener A.A. (1993). Safety factors in bone strength. Calcif. Tissue Int..

[94] Frost H.M. (2004). A 2003 update of bone physiology and Wolff’s Law for clinicians. Angle Orthod..

[95] Cilla M., Checa S., Duda G.N. (2017). Strain shielding inspired re-design of proximal femoral stems for total hip arthroplasty. J. Orthop. Res..

[96] Arabnejad S., Johnston B., Tanzer M., Pasini D. (2017). Fully porous 3D printed titanium femoral stem to reduce stress-shielding following total hip arthroplasty. J. Orthop. Res..

[97] Yan C., Hao L., Hussein A., Young P. (2015). Ti–6Al–4V triply periodic minimal surface structures for bone implants fabricated via selective laser melting. J. Mech. Behav. Biomed. Mater..

[98] Vaish A., Vaish R. (2018). 3D printing and its applications in Orthopedics. J. Clin. Orthop. Trauma.

[99] Eshkalak S.K., Ghomi E.R., Dai Y., Choudhury D., Ramakrishna S. (2020). The role of three-dimensional printing in healthcare and medicine. Mater. Des..

[100] Rehman M., Yanen W., Mushtaq R.T., Ishfaq K., Zahoor S., Ahmed A., Kumar M.S., Gueyee T., Rahman M.M., Sultana J. (2023). Additive manufacturing for biomedical applications: A review on classification, energy consumption, and its appreciable role since COVID-19 pandemic. Prog. Addit. Manuf..

[101] Geng J.P., Tan K.B., Liu G.R. (2001). Application of finite element analysis in implant dentistry: A review of the literature. J. Prosthet. Dent..

[102] Lang J.J., Bastian M., Foehr P., Seebach M., Weitz J., von Deimling C., Schwaiger B.J., Micheler C.M., Wilhelm N.J., Grosse C.U. (2021). Improving mandibular reconstruction by using topology optimization, patient specific design and additive manufacturing?-A biomechanical comparison against miniplates on human specimen. PLoS ONE.

[103] Moiduddin K., Mian S.H., Ameen W., Alkindi M., Ramalingam S., Alghamdi O. (2020). Patient-Specific Surgical Implant Using Cavity-Filled Approach for Precise and Functional Mandible Reconstruction. Appl. Sci..

[104] Xia Y., Feng Z.C., Li C., Wu H., Tang C., Wang L., Li H. (2020). Application of additive manufacturing in customized titanium mandibular implants for patients with oral tumors. Oncol. Lett..

[105] Nassehi A., Newman S., Dhokia V., Zhu Z., Asrai I.R. Using formal methods to model hybrid manufacturing processes. Proceedings of the 4th International Conference on Changeable, Agile, Reconfigurable and Virtual Production (CARV2011).

[106] Munir K., Biesiekierski A., Wen C., Li Y., Wen C. (2020). Selective laser melting in biomedical manufacturing. Metallic Biomaterials Processing and Medical Device Manufacturing.

[107] Attar H., Ehtemam-Haghighi S., Kent D., Wu X., Dargusch M.S. (2017). Comparative study of commercially pure titanium produced by laser engineered net shaping, selective laser melting and casting processes. Mater. Sci. Eng..

[108] Bose S., Ke D., Sahasrabudhe H., Bandyopadhyay A. (2018). Additive manufacturing of biomaterials. Prog. Mater. Sci..

[109] Wong K.V., Hernandez A. (2012). A Review of Additive Manufacturing. ISRN Mech. Eng..

[110] Popov V.V., Muller-Kamskii G., Kovalevsky A., Dzhenzhera G., Strokin E., Kolomiets A., Ramon J. (2018). Design and 3D-printing of titanium bone implants: Brief review of approach and clinical cases. Biomed. Eng. Lett..

[111] Wysocki B., Maj P., Sitek R., Buhagiar J., Kurzydłowski K., Święszkowski W. (2017). Laser and Electron Beam Additive Manufacturing Methods of Fabricating Titanium Bone Implants. Appl. Sci..

[112] Wehmöller M., Warnke P.H., Zilian C., Eufinger H. (2005). Implant design and production—A new approach by selective laser melting. Int. Congr. Ser..

[113] Krzyzanowski M., Svyetlichnyy D. (2021). A multiphysics simulation approach to selective laser melting modelling based on cellular automata and lattice Boltzmann methods. Comput. Part. Mech..

[114] Jahadakbar A., Shayesteh Moghaddam N., Amerinatanzi A., Dean D., Elahinia M., Naguib H.E. (2018). Mechanical evaluation of the SLM fabricated, stiffness-matched, mandibular bone fixation plates. SPIE 10596, Behavior and Mechanics of Multifunctional Materials and Composites XII.

[115] Murr L.E., Martinez E., Amato K.N., Gaytan S.M., Hernandez J., Ramirez D.A., Shindo P.W., Medina F., Wicker R.B. (2012). Fabrication of Metal and Alloy Components by Additive Manufacturing: Examples of 3D Materials Science. J. Mater. Res. Technol..

[116] Roudnicka M., Misurak M., Vojtech D. (2019). Differences in the Response of Additively Manufactured Titanium Alloy to Heat Treatment—Comparison between SLM and EBM. Manuf. Technol..

[117] Jahadakbar A., Moghaddam N.S., Amerinatanzi A., Dean D., Karaca H.E., Elahinia M. (2016). Finite Element Simulation and Additive Manufacturing of Stiffness-Matched NiTi Fixation Hardware for Mandibular Reconstruction Surgery. Bioeng.

[118] Ashby M.F. (2000). Metal Foams: A Design Guide.

[119] Qian L., Zhang H. (2011). Controlled freezing and freeze drying: A versatile route for porous and micro-/nano-structured materials. J. Chem. Technol. Biotechnol..

[120] Al-Ketan O., Lee D.-W., Abu Al-Rub R.K. (2021). Mechanical properties of additively-manufactured sheet-based gyroidal stochastic cellular materials. Addit. Manuf..

[121] Cansizoglu O., Harrysson O., Cormier D., West H., Mahale T. (2008). Properties of Ti–6Al–4V non-stochastic lattice structures fabricated via electron beam melting. Mater. Sci. Eng..

[122] Gibson L.J., Ashby M.F. (1988). Cellular Solids: Structure & Properties.

[123] Krishna B.V., Bose S., Bandyopadhyay A. (2007). Low stiffness porous Ti structures for load-bearing implants. Acta Biomater..

[124] Parthasarathy J., Starly B., Raman S., Christensen A. (2010). Mechanical evaluation of porous titanium (Ti6Al4V) structures with electron beam melting (EBM). J. Mech. Behav. Biomed. Mater..

[125] Hedayati R., Sadighi M., Mohammadi-Aghdam M., Hosseini-Toudeshky H. (2018). Comparison of elastic properties of open-cell metallic biomaterials with different unit cell types. J. Biomed. Mater. Res. B Appl. Biomater..

[126] Van Bael S., Chai Y.C., Truscello S., Moesen M., Kerckhofs G., Van Oosterwyck H., Kruth J.P., Schrooten J. (2012). The effect of pore geometry on the in vitro biological behavior of human periosteum-derived cells seeded on selective laser-melted Ti6Al4V bone scaffolds. Acta Biomater..

[127] Seto Y., Sharif Ullah A., Kubo A., D’Addona D.M., Teti R. On the Porous Structuring using Unit Cells. Proceedings of the 14th CIRP Conference on Intelligent Computation in Manufacturing Engineering.

[128] Bartolomeu F., Costa M.M., Alves N., Miranda G., Silva F.S. (2021). Selective Laser Melting of Ti6Al4V sub-millimetric cellular structures: Prediction of dimensional deviations and mechanical performance. J. Mech. Behav. Biomed. Mater..

[129] Dallago M., Raghavendra S., Luchin V., Zappini G., Pasini D., Benedetti M. (2021). The role of node fillet, unit-cell size and strut orientation on the fatigue strength of Ti-6Al-4V lattice materials additively manu-factured via laser powder bed fusion. Int. J. Fatigue.

[130] El-Sayed M.A., Essa K., Ghazy M., Hassanin H. (2020). Design optimization of additively manufactured titanium lattice structures for biomedical implants. Int. J. Adv. Manuf. Technol..

[131] Alabort E., Barba D., Reed R.C. (2019). Design of metallic bone by additive manufacturing. Scripta Mater..

[132] Ghouse S., Reznikov N., Boughton O.R., Babu S., Geoffrey Ng K.C., Blunn G., Cobb J.P., Stevens M.M., Jeffers J.R.T. (2019). The Design and In Vivo Testing of a Locally Stiffness-Matched Porous Scaffold. Appl. Mater. Today.

[133] Xiong Y.Z., Gao R.N., Zhang H., Dong L.L., Li J.T., Li X. (2020). Rationally designed functionally graded porous Ti6Al4V scaffolds with high strength and toughness built via selective laser melting for load-bearing orthopedic applications. J. Mech. Behav. Biomed. Mater..

[134] Soro N., Attar H., Wu X., Dargusch M.S. (2019). Investigation of the structure and mechanical properties of additively manufactured Ti-6Al-4V biomedical scaffolds designed with a Schwartz primitive unit-cell. Mater. Sci. Eng..

[135] du Plessis A., Yadroitsava I., Yadroitsev I. (2018). Ti6Al4V lightweight lattice structures manufactured by laser powder bed fusion for load-bearing applications. Opt. Laser Technol..

[136] Zhang B., Pei X., Zhou C., Fan Y., Jiang Q., Ronca A., D’Amora U., Chen Y., Li H., Sun Y. (2018). The biomimetic design and 3D printing of customized mechanical properties porous Ti6Al4V scaffold for load-bearing bone reconstruction. Mater. Des..

[137] Horn T.J., Harrysson O.L.A., Marcellin-Little D.J., West H.A., Lascelles B.D.X., Aman R. (2014). Flexural properties of Ti6Al4V rhombic dodecahedron open cellular structures fabricated with electron beam melting. Addit. Manuf..

[138] Di Caprio F., Franchitti S., Borrelli R., Bellini C., Di Cocco V., Sorrentino L. (2022). Ti-6Al-4V Octet-Truss Lattice Structures under Bending Load Conditions: Numerical and Experimental Results. Metals.

[139] Bellini C., Borrelli R., Di Cocco V., Franchitti S., Iacoviello F., Mocanu L.P., Sorrentino L. (2021). Failure energy and stiffness of titanium lattice specimens produced by electron beam melting process. Mater. Des. Process. Commun..

[140] Bellini C., Borrelli R., Di Cocco V., Franchitti S., Iacoviello F., Sorrentino L. (2021). Damage analysis of Ti6Al4V lattice structures manufactured by electron beam melting process subjected to bending load. Mater. Des. Process. Commun..

[141] Tüzemen M.Ç., Salamcı E., Ünal R. (2022). Investigation of the relationship between flexural modulus of elasticity and functionally graded porous structures manufactured by AM. Mater. Today Commun..

[142] Song J., Tang Q., Feng Q., Ma S., Guo F., Han Q. (2021). Investigation on the modelling approach for variable-density lattice structures fabricated using selective laser melting. Mater. Des..

[143] Xu Y., Han G., Huang G., Li T., Xia J., Guo D. (2023). Properties Evaluations of Topology Optimized Functionally Graded Lattice Structures Fabricated by Selective Laser Melting. Materials.

[144] Mahamood R.M., Akinlabi E.T. (2017). Types of Functionally Graded Materials and Their Areas of Application. Functionally Graded Materials.

[145] Mahmoud D., Elbestawi M.A. (2018). Selective laser melting of porosity graded lattice structures for bone implants. Int. J. Adv. Manuf. Technol..

[146] Zhao Z., Zhang X.S. (2021). Design of graded porous bone-like structures via a multi-material topology optimization approach. Struct. Multidiscip. Optim..

[147] Mukherjee S., Dhara S., Saha P. (2023). Design and Additive Manufacturing of Acetabular Implant with Continuously Graded Porosity. Bioengineering.

[148] Onal E., Frith J., Jurg M., Wu X., Molotnikov A. (2018). Mechanical Properties and In Vitro Behavior of Additively Manufactured and Functionally Graded Ti6Al4V Porous Scaffolds. Metals.

[149] Shi J., Yang J., Li Z., Zhu L., Li L., Wang X. (2017). Design and fabrication of graduated porous Ti-based alloy implants for biomedical applications. J. Alloy. Compd..

[150] Yang L., Mertens R., Ferrucci M., Yan C., Shi Y., Yang S. (2019). Continuous graded Gyroid cellular structures fabricated by selective laser melting: Design, manufacturing and mechanical properties. Mater. Des..

[151] Han C., Li Y., Wang Q., Wen S., Wei Q., Yan C., Hao L., Liu J., Shi Y. (2018). Continuous functionally graded porous titanium scaffolds manufactured by selective laser melting for bone implants. J. Mech. Behav. Biomed. Mater..

[152] Fousova M., Vojtech D., Kubasek J., Jablonska E., Fojt J. (2017). Promising characteristics of gradient porosity Ti-6Al-4V alloy prepared by SLM process. J. Mech. Behav. Biomed. Mater..

[153] Liu F., Zhang D.Z., Zhang P., Zhao M., Jafar S. (2018). Mechanical Properties of Optimized Diamond Lattice Structure for Bone Scaffolds Fabricated via Selective Laser Melting. Materials.

[154] Wang R., Ni S., Ma L., Li M. (2022). Porous construction and surface modification of titanium-based materials for osteogenesis: A review. Front. Bioeng Biotechnol..

[155] Grzeskowiak R.M., Schumacher J., Dhar M.S., Harper D.P., Mulon P.Y., Anderson D.E. (2020). Bone and Cartilage Interfaces With Orthopedic Implants: A Literature Review. Front. Surg..

[156] Lu X., Wu Z., Xu K., Wang X., Wang S., Qiu H., Li X., Chen J. (2021). Multifunctional Coatings of Titanium Implants Toward Promoting Osseointegration and Preventing Infection: Recent Developments. Front. Bioeng. Biotechnol..

[157] Xiao Y., Ding Y., Zhuang J., Sun R., Sun H., Bai L. (2022). Osteoimmunomodulation role of exosomes derived from immune cells on osseointegration. Front. Bioeng. Biotechnol..

[158] Bai L., Chen P., Zhao Y., Hang R., Yao X., Tang B., Liu C., Xiao Y., Hang R. (2021). A micro/nano-biomimetic coating on titanium orchestrates osteo/angio-genesis and osteoimmunomodulation for advanced osseointegration. Biomaterials.

[159] Colnot C., Romero D.M., Huang S., Rahman J., Currey J.A., Nanci A., Brunski J.B., Helms J.A. (2007). Molecular Analysis of Healing at a Bone-Implant Interface. J. Dent. Res..

[160] Sivaraj K.K., Adams R.H. (2016). Blood vessel formation and function in bone. Development.

[161] Franchi M., Fini M., Martini D., Orsini E., Leonardi L., Ruggeri A., Giavaresi G., Ottani V. (2005). Biological fixation of endosseous implants. Micron.

[162] Davies J.E. (2003). Understanding Peri-Implant Endosseous Healing. J. Dent. Educ..

[163] Branemark P.-I. (2005). The Osseointegration Book—From Calvarium to Calcaneus.

[164] Tan X.P., Tan Y.J., Chow C.S.L., Tor S.B., Yeong W.Y. (2017). Metallic powder-bed based 3D printing of cellular scaffolds for orthopaedic implants: A state-of-the-art review on manufacturing, topological design, mechanical properties and biocompatibility. Mater. Sci. Eng. C Mater. Biol. Appl..

[165] Bobbert F.S.L., Zadpoor A.A. (2017). Effects of bone substitute architecture and surface properties on cell response, angiogenesis, and structure of new bone. J. Mater. Chem. B.

[166] Xue W., Krishna B.V., Bandyopadhyay A., Bose S. (2007). Processing and biocompatibility evaluation of laser processed porous titanium. Acta Biomater..

[167] Rumpler M., Woesz A., Dunlop J.W., van Dongen J.T., Fratzl P. (2008). The effect of geometry on three-dimensional tissue growth. J. R. Soc. Interface.

[168] Zhang Y., Sun N., Zhu M., Qiu Q., Zhao P., Zheng C., Bai Q., Zeng Q., Lu T. (2022). The contribution of pore size and porosity of 3D printed porous titanium scaffolds to osteogenesis. Biomater. Adv..

[169] Dziaduszewska M., Zielinski A. (2021). Structural and Material Determinants Influencing the Behavior of Porous Ti and Its Alloys Made by Additive Manufacturing Techniques for Biomedical Applications. Materials.

[170] Ponader S., Wilmowsky C.V., Widenmayer M., Lutz R., Heinl P., Körner C., Singer R.F., Nkenke E., Neukam F.W., Schlegel K.A. (2010). In vivo performance of selective electron beam-melted Ti-6Al-4V structures. J. Biomed. Mater. Res. A.

[171] Taniguchi N., Fujibayashi S., Takemoto M., Sasaki K., Otsuki B., Nakamura T., Matsushita T., Kokubo T., Matsuda S. (2016). Effect of pore size on bone ingrowth into porous titanium implants fabricated by additive manufacturing: An in vivo experiment. Mater. Sci. Eng. C Mater. Biol. Appl..

[172] Watanabe R., Takahashi H., Matsugaki A., Uemukai T., Kogai Y., Imagama T., Yukata K., Nakano T., Sakai T. (2023). Novel nano-hydroxyapatite coating of additively manufactured three-dimensional porous implants improves bone ingrowth and initial fixation. J. Biomed. Mater. Res. B Appl. Biomater..

[173] Li S., Li X., Hou W., Nune K.C., Misra R.D.K., Correa-Rodriguez V.L., Guo Z., Hao Y., Yang R., Murr L.E. (2017). Fabrication of open-cellular (porous) titanium alloy implants: Osseointegration, vascularization and preliminary human trials. Sci. China Mater..

[174] Hofmann A.A., Bloebaum R.D., Bachus K.N. (1997). Progression of human bone ingrowth into porous-coated implants. Rate of bone ingrowth in humans. Acta Orthop. Scand..

[175] Kovacs A.E., Csernatony Z., Csamer L., Mehes G., Szabo D., Veres M., Braun M., Harangi B., Serban N., Zhang L. (2023). Comparative Analysis of Bone Ingrowth in 3D-Printed Titanium Lattice Structures with Different Patterns. Materials.

[176] de Wild M., Schumacher R., Mayer K., Schkommodau E., Thoma D., Bredell M., Kruse Gujer A., Gratz K.W., Weber F.E. (2013). Bone regeneration by the osteoconductivity of porous titanium implants manufactured by selective laser melting: A histological and micro computed tomography study in the rabbit. Tissue Eng. Part A.

[177] Roberts G.L., Pallister I. (2012). Finite element analysis in trauma & orthopaedics—An introduction to clinically relevant simulation & its limitations. Orthop. Trauma.

[178] Logan D.L. (2012). A First Course in the Finite Element Method.

[179] Baccouch M. (2021). A Brief Summary of the Finite Element Method for Differential Equations. Finite Element Methods and Their Applications.

[180] Ilavarasi P.U., Anburaian M. Design and Finite Element Analysis of Mandibular Prosthesis. Proceedings of the 2011 3rd International Conference on Electronics Computer Technology.

[181] Plumbridge W.J., Matela R.J., Westwater A., Finite Element Analysis (2003). Structural Integrity and Reliability in Electronics: Enhancing Performance in a Lead-Free Environment.

[182] Pidaparti R.M. (2017). Engineering Finite Element Analysis.

[183] Xiong Y., Han Z., Qin J., Dong L., Zhang H., Wang Y., Chen H., Li X. (2021). Effects of porosity gradient pattern on mechanical performance of additive manufactured Ti-6Al-4V functionally graded porous structure. Mater. Des..

[184] Zhao S., Li S.J., Hou W.T., Hao Y.L., Yang R., Misra R.D.K. (2016). The influence of cell morphology on the compressive fatigue behavior of Ti-6Al-4V meshes fabricated by electron beam melting. J. Mech. Behav. Biomed. Mater..

[185] Jin N., Yan Z., Wang Y., Cheng H., Zhang H. (2021). Effects of heat treatment on microstructure and mechanical properties of selective laser melted Ti-6Al-4V lattice materials. Int. J. Mech. Sci..

[186] Smith M., Guan Z., Cantwell W.J. (2013). Finite element modelling of the compressive response of lattice structures manufactured using the selective laser melting technique. Int. J. Mech. Sci..

[187] Panettieri E., Boissin E., Montemurro M., Catapano A., Jalocha D. (2022). On the accuracy of a homogenized continuum model of lattice structures in modal analyses. Mech. Adv. Mater. Struct..

[188] Dias M.R., Guedes J.M., Flanagan C.L., Hollister S.J., Fernandes P.R. (2014). Optimization of scaffold design for bone tissue engineering: A computational and experimental study. Med. Eng. Phys..

[189] Vollmer D., Meyer U., Joos U., Vegh A., Piffko J. (2000). Experimental and finite element study of a human mandible. J. Craniomaxillofac. Surg..

[190] Korioth T.W.P., Hannam A.G. (1994). Deformation of the Human Mandible During Simulated Tooth Clenching. J. Dent. Res..

[191] Luo D., Rong Q., Chen Q. (2017). Finite-element design and optimization of a three-dimensional tetrahedral porous titanium scaffold for the reconstruction of mandibular defects. Med. Eng. Phys..

[192] Yoon Y., Kim J.-e., Jung J., Oh S.-h., Noh G., Kwon Y.-D. (2021). Effect of mandibular contouring surgery on the stress distribution during various clenching tasks. J. Comput. Des. Eng..

[193] Burton H.E., Eisenstein N.M., Lawless B.M., Jamshidi P., Segarra M.A., Addison O., Shepherd D.E.T., Attallah M.M., Grover L.M., Cox S.C. (2019). The design of additively manufactured lattices to increase the functionality of medical implants. Mater. Sci. Eng. C Mater. Biol. Appl..

[194] Ji B., Wang C., Liu L., Long J., Tian W., Wang H. (2010). A biomechanical analysis of titanium miniplates used for treatment of mandibular symphyseal fractures with the finite element method. Oral Surg. Oral Med. Oral Pathol. Oral Radiol. Endod..

[195] Zhong S., Shi Q., Sun Y., Yang S., Van Dessel J., Gu Y., Chen X., Lubbers H.T., Politis C. (2021). Biomechanical comparison of locking and non-locking patient-specific mandibular reconstruction plate using finite element analysis. J. Mech. Behav. Biomed. Mater..

[196] Jesus G.P.D., Vaz L.G., Gabrielli M.F.R., Passeri L.A., Oliveira T.V., Noritomi P.Y., Jürgens P. (2014). Finite element evaluation of three methods of stable fixation of condyle base fractures. Int. J. Oral Maxillofac. Surg..

[197] Ghosh A., Kumar A., Wang X., Kietzig A.-M., Brochu M. (2022). Analysis of the effect of surface morphology on tensile behavior of LPBF SS316L microstruts. Mater. Sci. Eng..

[198] Yánez A., Fiorucci M.P., Cuadrado A., Martel O., Monopoli D. (2020). Surface roughness effects on the fatigue behaviour of gyroid cellular structures obtained by additive manufacturing. Int. J. Fatigue.

[199] Kondo S., Katsuta H., Akizuki A., Kurihara Y., Kamatani T., Yaso A., Nagasaki M., Shimane T., Shirota T. (2015). Computer-Assisted Surgery for Mandibular Reconstruction Using a Patient-Specific Titanium Mesh Tray and Particulate Cancellous Bone and Marrow. Case Rep. Clin. Med..

[200] Lee H., Park S., Noh G. (2019). Biomechanical analysis of 4 types of short dental implants in a resorbed mandible. J. Prosthet. Dent..

[201] Malekpour Z., Sarkarat F., Hooshangi H. (2014). Mandibular Reconstruction Using Custom-Made Titanium Mesh Tray and Autogenous Bone Graft—A Case Report. Thrita.

[202] Mounir M., Abou-ElFetouh A., ElBeialy W., Mounir R. (2020). Patient-specific alloplastic endoprosthesis for reconstruction of the mandible following segmental resection: A case series. J. Craniomaxillofac. Surg..

[203] Park J.H., Odkhuu M., Cho S., Li J., Park B.Y., Kim J.W. (2020). 3D-printed titanium implant with pre-mounted dental implants for mandible reconstruction: A case report. Maxillofac. Plast. Reconstr. Surg..

[204] Mommaerts M.Y. (2016). Guidelines for patient-specific jawline definition with titanium implants in esthetic, deformity, and malformation surgery. Ann. Maxillofac. Surg..

[205] Snell R., Tammas-Williams S., Chechik L., Lyle A., Hernández-Nava E., Boig C., Panoutsos G., Todd I. (2019). Methods for Rapid Pore Classification in Metal Additive Manufacturing. J. Miner. Met. Mater. Soc..

[206] Zhang B., Li Y., Bai Q. (2017). Defect Formation Mechanisms in Selective Laser Melting: A Review. Chin. J. Mech. Eng..

[207] Khairallah S.A., Anderson A.T., Rubenchik A., King W.E. (2016). Laser powder-bed fusion additive manufacturing: Physics of complex melt flow and formation mechanisms of pores, spatter, and denudation zones. Acta Mater..

[208] Kan W.H., Gao M., Zhang X., Liang E., Chiu N.S.L., Lim C.V.S., Huang A. (2022). The influence of porosity on Ti-6Al-4V parts fabricated by laser powder bed fusion in the pursuit of process efficiency. Int. J. Adv. Manuf. Technol..

[209] Park J.B., Lakes R.S. (2007). Metallic Implant Materials. Biomaterials: An Introduction.

[210] Ge J., Huang Q., Wang Y., Zhang C., Liu Q., Lu Z., Yin S. (2023). Microstructural optimization and mechanical enhancement of SLM Ti6Al4V TPMS scaffolds through vacuum annealing treatment. J. Alloys Compd..

[211] Yan X., Lupoi R., Wu H., Ma W., Liu M., O’Donnell G., Yin S. (2019). Effect of hot isostatic pressing (HIP) treatment on the compressive properties of Ti6Al4V lattice structure fabricated by selective laser melting. Mater. Lett..

[212] Gong H., Rafi K., Gu H., Janaki Ram G.D., Starr T., Stucker B. (2015). Influence of defects on mechanical properties of Ti–6Al–4V components produced by selective laser melting and electron beam melting. Mater. Des..

[213] Murr L.E., Gaytan S.M., Ramirez D.A., Martinez E., Hernandez J., Amato K.N., Shindo P.W., Medina F.R., Wicker R.B. (2012). Metal Fabrication by Additive Manufacturing Using Laser and Electron Beam Melting Technologies. J. Mater. Sci. Technol..

[214] Yadroitsev I., Krakhmalev P., Yadroitsava I., Du Plessis A. (2017). Qualification of Ti6Al4V ELI Alloy Produced by Laser Powder Bed Fusion for Biomedical Applications. J. Miner. Met. Mater. Soc..

[215] Thijs L., Verhaeghe F., Craeghs T., Humbeeck J.V., Kruth J.-P. (2010). A study of the microstructural evolution during selective laser melting of Ti–6Al–4V. Acta Mater..

[216] Yadroitsev I., Krakhmalev P., Yadroitsava I. (2015). Hierarchical design principles of selective laser melting for high quality metallic objects. Addit. Manuf..

[217] Cunningham R., Narra S.P., Montgomery C., Beuth J., Rollett A.D. (2017). Synchrotron-Based X-ray Microtomography Characterization of the Effect of Processing Variables on Porosity Formation in Laser Power-Bed Additive Manufacturing of Ti-6Al-4V. J. Miner. Met. Mater. Soc..

[218] Kan W.H., Chiu L.N.S., Lim C.V.S., Zhu Y., Tian Y., Jiang D., Huang A. (2022). A critical review on the effects of process-induced porosity on the mechanical properties of alloys fabricated by laser powder bed fusion. J. Mater. Sci..

[219] Montalbano T., Briggs B.N., Waterman J.L., Nimer S., Peitsch C., Sopcisak J., Trigg D., Storck S. (2021). Uncovering the coupled impact of defect morphology and microstructure on the tensile behavior of Ti-6Al-4V fabricated via laser powder bed fusion. J. Mater. Process. Technol..

[220] du Plessis A., Yadroitsava I., Yadroitsev I. (2020). Effects of defects on mechanical properties in metal additive manufacturing: A review focusing on X-ray tomography insights. Mater. Des..

[221] Cao S., Chen Z., Lim C.V.S., Yang K., Jia Q., Jarvis T., Tomus D., Wu X. (2017). Defect, Microstructure, and Mechanical Property of Ti-6Al-4V Alloy Fabricated by High-Power Selective Laser Melting. J. Miner. Met. Mater. Soc..

[222] Pal S., Gubeljak N., Hudák R., Lojen G., Rajťúková V., Brajlih T., Drstvenšek I. (2020). Evolution of the metallurgical properties of Ti-6Al-4V, produced with different laser processing parameters, at constant energy density in selective laser melting. Results Phys..

[223] Antony K., Arivazhagan N. (2015). Studies on energy penetration and Marangoni effect during laser melting process. J. Eng. Sci. Technol..

[224] Svenungsson J., Choquet I., Kaplan A.F.H. (2015). Laser Welding Process—A Review of Keyhole Welding Modelling. Phys. Procedia.

[225] Voznesenskaya A.A., Zhdanov A.V., Raznoschikov A.S. (2021). Evolution of porosity depending on SLM mode and subsequent HIP processing. J. Phys. Conf. Ser..

[226] Ransenigo C., Tocci M., Palo F., Ginestra P., Ceretti E., Gelfi M., Pola A. (2022). Evolution of Melt Pool and Porosity During Laser Powder Bed Fusion of Ti6Al4V Alloy: Numerical Modelling and Experimental Validation. Lasers Manuf. Mater. Process..

[227] Salem H., Carter L.N., Attallah M.M., Salem H.G. (2019). Influence of processing parameters on internal porosity and types of defects formed in Ti6Al4V lattice structure fabricated by selective laser melting. Mater. Sci. Eng..

[228] Campoli G., Borleffs M.S., Amin Yavari S., Wauthle R., Weinans H., Zadpoor A.A. (2013). Mechanical properties of open-cell metallic biomaterials manufactured using additive manufacturing. Mater. Des..

[229] du Plessis A., Razavi S.M.J., Berto F. (2020). The effects of microporosity in struts of gyroid lattice structures produced by laser powder bed fusion. Mater. Des..

[230] Hu Y.N., Wu S.C., Withers P.J., Zhang J., Bao H.Y.X., Fu Y.N., Kang G.Z. (2020). The effect of manufacturing defects on the fatigue life of selective laser melted Ti-6Al-4V structures. Mater. Des..

[231] Leuders S., Thöne M., Riemer A., Niendorf T., Tröster T., Richard H.A., Maier H.J. (2013). On the mechanical behaviour of titanium alloy TiAl6V4 manufactured by selective laser melting: Fatigue resistance and crack growth performance. Int. J. Fatigue.

[232] Shipley H., McDonnell D., Culleton M., Coull R., Lupoi R., O’Donnell G., Trimble D. (2018). Optimisation of process parameters to address fundamental challenges during selective laser melting of Ti-6Al-4V: A review. Int. J. Mach. Tools Manuf..

[233] Wickmann C., Benz C., Heyer H., Witte-Bodnar K., Schafer J., Sander M. (2021). Internal Crack Initiation and Growth Starting from Artificially Generated Defects in Additively Manufactured Ti6Al4V Specimen in the VHCF Regime. Materials.

[234] Ahmadi S.M., Kumar R., Borisov E.V., Petrov R., Leeflang S., Li Y., Tumer N., Huizenga R., Ayas C., Zadpoor A.A. (2019). From microstructural design to surface engineering: A tailored approach for improving fatigue life of additively manufactured meta-biomaterials. Acta Biomater..

[235] Chern A.H., Nandwana P., Yuan T., Kirka M.M., Dehoff R.R., Liaw P.K., Duty C.E. (2019). A review on the fatigue behavior of Ti-6Al-4V fabricated by electron beam melting additive manufacturing. Int. J. Fatigue.

[236] Liu Y.J., Ren D.C., Li S.J., Wang H., Zhang L.C., Sercombe T.B. (2020). Enhanced fatigue characteristics of a topology-optimized porous titanium structure produced by selective laser melting. Addit. Manuf..

[237] Zhao S., Li S.J., Wang S.G., Hou W.T., Li Y., Zhang L.C., Hao Y.L., Yang R., Misra R.D.K., Murr L.E. (2018). Compressive and fatigue behavior of functionally graded Ti-6Al-4V meshes fabricated by electron beam melting. Acta Mater..

[238] Ren D., Li S., Wang H., Hou W., Hao Y., Jin W., Yang R., Misra R.D.K., Murr L.E. (2019). Fatigue behavior of Ti-6Al-4V cellular structures fabricated by additive manufacturing technique. J. Mater. Sci. Technol..

[239] Xiong Y., Wang W., Gao R., Zhang H., Dong L., Qin J., Wang B., Jia W., Li X. (2020). Fatigue behavior and osseointegration of porous Ti-6Al-4V scaffolds with dense core for dental application. Mater. Des..

[240] Antonysamy A.A., Meyer J., Prangnell P.B. (2013). Effect of build geometry on the β-grain structure and texture in additive manufacture of Ti6Al4V by selective electron beam melting. Mater. Charact..

[241] Kumar P., Prakash O., Ramamurty U. (2018). Micro-and meso-structures and their influence on mechanical properties of selectively laser melted Ti-6Al-4V. Acta Mater..

[242] Tong J., Bowen C.R., Persson J., Plummer A. (2016). Mechanical properties of titanium-based Ti–6Al–4V alloys manufactured by powder bed additive manufacture. Mater. Sci. Tech. Ser..

[243] Wycisk E., Solbach A., Siddique S., Herzog D., Walther F., Emmelmann C. (2014). Effects of Defects in Laser Additive Manufactured Ti-6Al-4V on Fatigue Properties. Phys. Procedia.

[244] Gao H.-J., Zhang Y.-D., Wu Q., Song J. (2017). Experimental Investigation on the Fatigue Life of Ti-6Al-4V Treated by Vibratory Stress Relief. Metals.

[245] Moussaoui K., Mousseigne M., Senatore J., Chieragatti R., Lamesle P. (2015). Influence of Milling on the Fatigue Lifetime of a Ti6Al4V Titanium Alloy. Metals.

[246] Belan J., Kuchariková L., Tillová E., Chalupová M. (2019). Three-Point Bending Fatigue Test of TiAl6V4 Titanium Alloy at Room Temperature. Adv. Mater. Sci. Eng..

[247] Dallago M., Fontanari V., Torresani E., Leoni M., Pederzolli C., Potrich C., Benedetti M. (2018). Fatigue and biological properties of Ti-6Al-4V ELI cellular structures with variously arranged cubic cells made by selective laser melting. J. Mech. Behav. Biomed. Mater..

[248] Yavari S.A., Ahmadi S.M., Wauthle R., Pouran B., Schrooten J., Weinans H., Zadpoor A.A. (2015). Relationship between unit cell type and porosity and the fatigue behavior of selective laser melted meta-biomaterials. J. Mech. Behav. Biomed. Mater..

[249] As S., Skallerud B., Tveiten B. (2008). Surface roughness characterization for fatigue life predictions using finite element analysis. Int. J. Fatigue.

[250] Deligianni D.D., Katsala N., Ladas S., Biomaterials S.D. (2001). Effect of surface roughness of the titanium alloy Ti–6Al–4V on human bone marrow cell response and on protein adsorption. Biomaterials.

[251] Palanivel S., Dutt A.K., Faierson E.J., Mishra R.S. (2016). Spatially dependent properties in a laser additive manufactured Ti–6Al–4V component. Mater. Sci. Eng..

[252] Hernández-Nava E., Smith C.J., Derguti F., Tammas-Williams S., Leonard F., Withers P.J., Todd I., Goodall R. (2016). The effect of defects on the mechanical response of Ti-6Al-4V cubic lattice structures fabricated by electron beam melting. Acta Mater..

[253] Xiao L., Li S., Song W., Xu X., Gao S. (2020). Process-induced geometric defect sensitivity of Ti–6Al–4V lattice structures with different mesoscopic topologies fabricated by electron beam melting. Mater. Sci. Eng..

[254] Alghamdi A., Downing D., McMillan M., Brandt M., Qian M., Leary M. (2019). Experimental and numerical assessment of surface roughness for Ti6Al4V lattice elements in selective laser melting. Int. J. Adv. Manuf. Technol..

[255] Wang D., Lv J., Wei X., Lu D., Chen C. (2023). Study on Surface Roughness Improvement of Selective Laser Melted Ti6Al4V Alloy. Crystals.

[256] Luis Pérez C.J., Vivancos Calvet J., Sebastián Pérez M.A. (2001). Geometric roughness analysis in solid free-form manufacturing processes. J. Mater. Process. Technol..

[257] Pyka G., Kerckhofs G., Papantoniou I., Speirs M., Schrooten J., Wevers M. (2013). Surface Roughness and Morphology Customization of Additive Manufactured Open Porous Ti6Al4V Structures. Materials.

[258] Tian Y., Tomus D., Rometsch P., Wu X. (2017). Influences of processing parameters on surface roughness of Hastelloy X produced by selective laser melting. Addit. Manuf..

[259] Xiang Y., Zhang S., Wei Z., Li J., Wei P., Chen Z., Yang L., Jiang L. (2018). Forming and defect analysis for single track scanning in selective laser melting of Ti6Al4V. Appl. Phys. A.

[260] Ahmadi S.M., Hedayati R., Li Y., Lietaert K., Tümer N., Fatemi A., Rans C.D., Pouran B., Weinans H., Zadpoor A.A. (2018). Fatigue performance of additively manufactured meta-biomaterials: The effects of topology and material type. Acta Biomater..

[261] Oosterbeek R.N., Jeffers J.R.T. (2022). StrutSurf: A tool for analysis of strut morphology and surface roughness in additively manufactured lattices. SoftwareX.

[262] Cao F., Zhang T., Ryder M.A., Lados D.A. (2018). A Review of the Fatigue Properties of Additively Manufactured Ti-6Al-4V. J. Miner. Met. Mater. Soc..

[263] Hallab N.J. (2009). A review of the biologic effects of spine implant debris: Fact from fiction. SAS J..

[264] Oliveira L.Y., Kuromoto N.K., Siqueira C.J. (2014). Treating orthopedic prosthesis with diamond-like carbon: Minimizing debris in Ti6Al4V. J. Mater. Sci. Mater. Med..

[265] Goodman S.B., Gallo J., Gibon E., Takagi M. (2020). Diagnosis and management of implant debris-associated inflammation. Expert Rev. Med. Devices.

[266] Kasperovich G., Hausmann J. (2015). Improvement of fatigue resistance and ductility of TiAl6V4 processed by selective laser melting. J. Mater. Process. Technol..

[267] Tang M., Pistorius P.C., Beuth J.L. (2017). Prediction of lack-of-fusion porosity for powder bed fusion. Addit. Manuf..

[268] Khairallah S.A., Martin A.A., Lee J.R.I., Guss G., Calta N.P., Hammons J.A., Nielsen M.H., Chaput K., Schwalbach E., Shah M.N. (2020). Controlling interdependent meso-nanosecond dynamics and defect generation in metal 3D printing. Science.

[269] du Plessis A., Yadroitsev I., Yadroitsava I., Le Roux S.G. (2018). X-Ray Microcomputed Tomography in Additive Manufacturing: A Review of the Current Technology and Applications. 3D Print Addit. Manuf..

[270] Yadroitsev I., Smurov I. (2011). Surface Morphology in Selective Laser Melting of Metal Powders. Phys. Procedia.

[271] Shiomi M., Osakada K., Nakamura K., Yamashita T., Abe F. (2004). Residual Stress within Metallic Model Made by Selective Laser Melting Process. CIRP Ann..

[272] Matsumoto Y., Hashimoto F., Lahoti G. (1999). Surface Integrity Generated by Precision Hard Turning. CIRP Ann..

[273] Li C., Liu Z.Y., Fang X.Y., Guo Y.B. (2018). Residual Stress in Metal Additive Manufacturing. Proc. Cirp..

[274] Yuan W., Hou W., Li S., Hao Y., Yang R., Zhang L.-C., Zhu Y. (2018). Heat treatment enhancing the compressive fatigue properties of open-cellular Ti-6Al-4V alloy prototypes fabricated by electron beam melting. J. Mater. Sci. Technol..

[275] Tammas-Williams S., Withers P.J., Todd I., Prangnell P.B. (2016). The Effectiveness of Hot Isostatic Pressing for Closing Porosity in Titanium Parts Manufactured by Selective Electron Beam Melting. Metall. Mater. Trans. A.

[276] Atkinson H.V., Davies S. (2000). Fundamental aspects of hot isostatic pressing: An overview. Metall. Mater. Trans. A.

[277] Guo R.P., Cheng M., Zhang C.J., Qiao J.W., Cai C., Wang Q.J., Xu D.S., Xu L., Yang R., Shi Y.S. (2023). Achieving superior fatigue strength in a powder-metallurgy titanium alloy via in-situ globularization during hot isostatic pressing. Scripta Mater.

[278] Delo D.P., Piehler H.R. (1999). Early stage consolidation mechanisms during hot isostatic pressing of Ti–6Al–4V powder compacts. Acta Mater..

[279] Aslan N., Aksakal B., Findik F. (2021). Fabrication of porous-Ti6Al4V alloy by using hot pressing technique and Mg space holder for hard-tissue biomedical applications. J. Mater. Sci. Mater. Med..

[280] Liu L., Zheng H., Deng C. (2019). Influence of HIP Treatment on Mechanical Properties of Ti6Al4V Scaffolds Prepared by L-PBF Process. Metals.

[281] du Plessis A., Glaser D., Moller H., Mathe N., Tshabalala L., Mfusi B., Mostert R. (2019). Pore Closure Effect of Laser Shock Peening of Additively Manufactured AlSi10Mg. 3D Print. Addit. Manuf..

[282] du Plessis A., Rossouw P. (2015). Investigation of Porosity Changes in Cast Ti6Al4V Rods After Hot Isostatic Pressing. J. Mater. Eng. Perform..

[283] Aguado-Montero S., Navarro C., Vázquez J., Lasagni F., Slawik S., Domínguez J. (2022). Fatigue behaviour of PBF additive manufactured TI6AL4V alloy after shot and laser peening. Int. J. Fatigue.

[284] Jamshidi P., Aristizabal M., Kong W., Villapun V., Cox S.C., Grover L.M., Attallah M.M. (2020). Selective Laser Melting of Ti-6Al-4V: The Impact of Post-processing on the Tensile, Fatigue and Biological Properties for Medical Implant Applications. Materials.

[285] Cox S.C., Jamshidi P., Eisenstein N.M., Webber M.A., Burton H., Moakes R.J.A., Addison O., Attallah M., Shepherd D.E.T., Grover L.M. (2017). Surface Finish has a Critical Influence on Biofilm Formation and Mammalian Cell Attachment to Additively Manufactured Prosthetics. ACS BioMater. Sci. Eng..

[286] Pattabi M., Ramakrishna K. (2008). Effect of mechanical cutting and polishing on the shape memory transformation behavior of NiTi alloy. Mater. Sci. Eng..

[287] Dong G., Marleau-Finley J., Zhao Y.F. (2019). Investigation of electrochemical post-processing procedure for Ti-6Al-4V lattice structure manufactured by direct metal laser sintering (DMLS). Int. J. Adv. Manuf. Technol..

[288] Kuhn A. (2004). Electropolishing of titanium and its alloys. Met. Finish..

[289] Zhang Y., Li J., Che S., Tian Y. (2019). Electrochemical Polishing of Additively Manufactured Ti–6Al–4V Alloy. Met. Mater. Int..

[290] Tsoeunyane G.M., Mathe N., Tshabalala L., Makhatha M.E., Luo Z. (2022). Electropolishing of Additively Manufactured Ti-6Al-4V Surfaces in Nontoxic Electrolyte Solution. Adv. Mater. Sci. Eng..

[291] Łyczkowska E., Szymczyk P., Dybała B., Chlebus E. (2014). Chemical polishing of scaffolds made of Ti–6Al–7Nb alloy by additive manufacturing. Arch. Civ. Mech. Eng..

[292] Hung W. (2021). Postprocessing of Additively Manufactured Metal Parts. J. Mater. Eng. Perform..

[293] Pyka G., Burakowski A., Kerckhofs G., Moesen M., Van Bael S., Schrooten J., Wevers M. (2012). Surface Modification of Ti6Al4V Open Porous Structures Produced by Additive Manufacturing. Adv. Eng. Mater..

[294] Berger M.B., Jacobs T.W., Boyan B.D., Schwartz Z. (2020). Hot isostatic pressure treatment of 3D printed Ti6Al4V alters surface modifications and cellular response. J. Biomed. Mater. Res. B Appl. Biomater..

[295] Disa J.J., Cordeiro P.G. (2000). Mandible reconstruction with microvascular surgery. Semin. Surg. Oncol..

[296] Shenaq S.M., Klebuc M.J.A. (1994). TMJ reconstruction during vascularized bone graft transfer to the mandible. Microsurgery.

[297] Park J.H., Jo E., Cho H., Kim H.J. (2017). Temporomandibular joint reconstruction with alloplastic prosthesis: The outcomes of four cases. Maxillofac. Plast. Reconstr. Surg..

[298] Mercuri L.G. (2018). Costochondral Graft Versus Total Alloplastic Joint for Temporomandibular Joint Reconstruction. Oral Maxillofac. Surg. Clin. N. Am..

[299] Emshoff R., Bertram A., Hupp L., Rudisch A. (2021). Condylar erosion is predictive of painful closed lock of the temporomandibular joint: A magnetic resonance imaging study. Head Face Med..

[300] Benady A., Meyer S.J., Golden E., Dadia S., Katarivas Levy G. (2023). Patient-specific Ti-6Al-4V lattice implants for critical-sized load-bearing bone defects reconstruction. Mater. Des..

[301] Cordey J., Borgeaud M., Perren S.M. (2000). Force transfer between the plate and the bone: Relative importance of the bending stiffness of the screws and the friction between plate and bone. Injury.

[302] Fukuda A., Takemoto M., Saito T., Fujibayashi S., Neo M., Pattanayak D.K., Matsushita T., Sasaki K., Nishida N., Kokubo T. (2011). Osteoinduction of porous Ti implants with a channel structure fabricated by selective laser melting. Acta Biomater..

[303] Bohara S., Suthakorn J. (2022). Surface coating of orthopedic implant to enhance the osseointegration and reduction of bacterial colonization: A review. Biomater. Res..

[304] Zimmerli W., Trampuz A., Ochsner P.E. (2004). Prosthetic-Joint Infections. N. Engl. J. Med..

[305] du Plessis A., Yadroitsava I., Kouprianoff D., Yadroitsev I. (2018). Numerical and experimental study of the effect of artificial porosity in a lattice structure manufactured by laser based powder bed fusion. Solid Freeform Fabrication Symposium—An Additive Manufacturing Conference.

[306] Al-Sukhun J., Helenius M., Lindqvist C., Kelleway J. (2006). Biomechanics of the Mandible Part I: Measurement of Mandibular Functional Deformation Using Custom-Fabricated Displacement Transducers. J. Oral Maxillofac. Surg..

